# Interaction of the putative tyrosine recombinases RipX (UU145), XerC (UU222), and CodV (UU529) of *Ureaplasma parvum* serovar 3 with specific DNA

**DOI:** 10.1111/1574-6968.12077

**Published:** 2013-01-31

**Authors:** Carl-Ulrich R Zimmerman, Renate Rosengarten, Joachim Spergser

**Affiliations:** Institute of Bacteriology, Mycology and Hygiene, University of Veterinary Medicine ViennaVienna, Austria

**Keywords:** Ureaplasma, tyrosine recombinase, protein–DNA interaction, electrophoretic mobility shift assay, phase variation, dif site

## Abstract

Phase variation of two loci (‘*mba* locus’ and ‘UU172 phase-variable element’) in *Ureaplasma parvum* serovar 3 has been suggested as result of site-specific DNA inversion occurring at short inverted repeats. Three potential tyrosine recombinases (RipX, XerC, and CodV encoded by the genes UU145, UU222, and UU529) have been annotated in the genome of *U. parvum* serovar 3, which could be mediators in the proposed recombination event. We document that only orthologs of the gene *xerC* are present in all strains that show phase variation in the two loci. We demonstrate *in vitro* binding of recombinant maltose-binding protein fusions of XerC to the inverted repeats of the phase-variable loci, of RipX to a direct repeat that flanks a 20-kbp region, which has been proposed as putative pathogenicity island, and of CodV to a putative *dif* site. Co-transformation of the model organism *Mycoplasma pneumoniae* M129 with both the ‘*mba* locus’ and the recombinase gene *xerC* behind an active promoter region resulted in DNA inversion in the ‘*mba* locus’. Results suggest that XerC of *U. parvum* serovar 3 is a mediator in the proposed DNA inversion event of the two phase-variable loci.

## Introduction

*Ureaplasma (U.) parvum* and *U. urealyticum* are commensals and potential pathogens of the human genital tract. The organism has been associated with nongonococcal, nonchlamydial urethritis in men, chorioamnionitis in pregnant women as well as bronchopulmonary dysplasia in newborn infants (Waites *et al*., 2005). Fourteen serovars have been identified, of which serovars 1, 3, 6, and 14 belong to the *U. parvum* species and the remaining to the *U. urealyticum* species (Robertson *et al*., 2002). The genomes of all 14 described serovars have been sequenced (Glass *et al*., 2000; Paralanov *et al*., 2012).

**Fig. 1:**
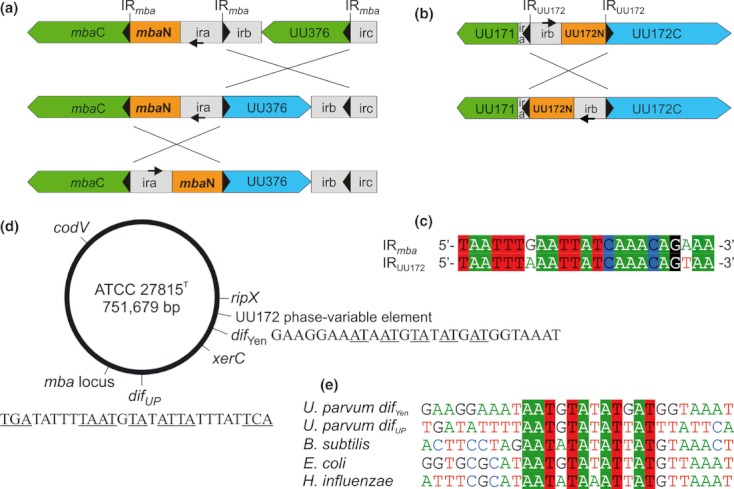
Phase-variable loci in *Ureaplasma parvum* serovar 3 and features of the potential *dif*_*UP*_ site. Schematic illustrations of site-specific DNA inversion events within the ‘*mba* locus’ (a) and the ‘UU172 phase-variable element’ (b). Captions and labeling: *mba*N, non-repetitive region of *mba*; *mba*C, repetitive region of *mba*; UU172N, N-terminal encoding region of UU172; UU172C: C-terminal encoding region of UU172; IR_*mba*_ and IR_UU__172_, inverted repeats; ira, irb, and irc, intergenic regions within the loci; black triangle, short inverted repeat; black arrow, putative promoter region; and black cross, DNA inversion. (c) Partial alignment of the inverted repeats IR_UU__172_ and IR_*mba*_ of the ‘UU172 phase-variable element’ and the ‘*mba* locus’. (d) Circular chromosome of *U. parvum* serovar 3 type strain ATCC 27815^T^ and locations of the three recombinase genes, the two potential *dif* sites *dif*_Yen_ (Yen *et al*., 2002) and *dif*_*UP*_, the ‘*mba* locus’, and the ‘UU172 phase-variable element’. Nucleotides appearing as palindrome within potential *dif* sites are underlined. (e) Alignment of the postulated *dif* sites from *U. parvum* with *dif* sites from *Bacillus subtilis*, *Escherichia coli,* and *Haemophilus influenzae*.

Both species express a distinct immunodominant, size- and phase-variable surface protein, the multiple-banded antigen, whose gene is one member of a paralogous gene family dispersed throughout the chromosome (Teng *et al*., 1994; Zheng *et al*., 1994, 1995; Glass *et al*., 2000; Monecke *et al*., 2003). In *U. parvum* serovar 3, two loci (‘*mba* locus’ and ‘UU172 phase-variable element’) have been identified that undergo high-frequency phase variation that is achieved by site-specific DNA inversions at short inverted repeats (Fig. 1a and b). Phase variation between UU375 (GenBank: AAF30784.1) (*mba* for multiple banded antigen) and UU376 (GenBank: AAF30785.1) (*upvmp* for Ureaplasma phase-variable membrane protein) is believed to be the result of site-specific DNA recombination at the inverted repeats 5′-ATTTG AATTATCAAACAGAAAAAG-3′ and occurs when the ORFs are oriented in opposite directions (Zimmerman *et al*., 2009). The second, more conserved phase-variable locus among the *Ureaplasma* species ‘UU172 phase-variable element’, like the ‘*mba* locus’ of *U. parvum* serovar 3, comprises two coding sequences (UU172 and UU171), which are oriented in opposite direction. Two inverted repeats (5′-ATA**ATTT**A**AATTATCAAACAG**T**AA**CTTTTGAACAAGTTCCT-3′), one located in the 5′ sequence of UU172 and another in the intergenic spacer region between UU172 and UU171, share partial identity (letters in bold and Fig. 1c) to the inverted repeats of the ‘*mba* locus’. It is believed that phase-variable expression of the UU172 element is governed by site-specific DNA inversion analogous to that occurring in the ‘*mba* locus’ (Zimmerman *et al*., 2011).

Three potential tyrosine recombinases (RipX, XerC, and CodV) have been annotated in the genome of *U. parvum* serovar 3 (Glass *et al*., 2000). To date, these three proteins have neither been functionally characterized nor have their binding sites been determined. Of the three genes, *ripX* (UU145) is located near the ‘*mba* locus’ in the ATCC strains of serovars 4, 5, 6, 7, 8, 9, 10, 11, and 12, suggesting an involvement of RipX in the site-specific recombination event in the ‘*mba* locus’. The gene is, however, also located at the boundary of a 20-kbp genomic region that has previously been proposed as a potential pathogenicity island (Momynaliev *et al*., 2007). Absence of this 20-kb region and *ripX* has been documented for serovars 1, 2, 13, 14 (Paralanov *et al*., 2012), and clinical isolates of serovars 1 and 6 (Momynaliev *et al*., 2007), which questions the protein's involvement in the site-specific recombination event of the phase-variable loci. Two 22-bp direct repeats (5′-TAATCGTGATTATTGAACCTTG-3′) that are located at the boundaries of the 20-kb region in serovar 10 suggest that the region has been acquired by horizontal gene transfer. Mobility of the region can be inferred from its different location in serovar 3, where the region disrupts a gene encoding a putative membrane protein of the ‘UU172 phase-variable element’ (Zimmerman *et al*., 2011).

All three potential recombinases of *U. parvum* possess typical genetic features that place them in the family of tyrosine recombinases (Fig. S1), such as the four conserved residues in the catalytic C-terminal half of the protein, which occur in the order Arg, His–X–X–Arg, and Tyr, with Tyr closest to the C-terminus (Argos *et al*., 1986; Esposito & Scocca, 1997). They also encode the two conserved, polar residues of the described DNA- or core-binding domain found in lambda (λ-)Int, designated T96 and S139 (Swalla *et al*., 2003).

Recombinases belonging to the tyrosine family are integrases that recombine DNA duplexes by executing two consecutive strand breakage and rejoining steps and a topoisomerization of their substrate (Esposito & Scocca, 1997). The first member of this family that was described is the λ-Int protein, which promotes integration and excision of the phage genome from that of the host (Nash, 1981). Other family members related to the λ-Int, such as the Flp from the yeast 2μ plasmid, the XerC/D of *Escherichia* (*E*.) *coli*, the Cre recombinase of phage P1, the HvsR of *Mycoplasma* (*M*.) *pulmonis,* and the Xer1 of *M. agalactiae,* function in the amplification/maintenance of plasmid copy number (Hoess *et al*., 1984), the elimination of chromosome dimers from replicated chromosomes (Hayes & Sherratt, 1997), the cyclization of virion DNA and the life cycle of temperate phages (Sternberg *et al*., 1986), the alteration of the type I restriction modification system and of cell-surface components (Sitaraman *et al*., 2002), and in phase variation of membrane proteins (Czurda *et al*., 2010), respectively. In *E. coli*, the proteins XerC and XerD (CodV and RipX in *Bacillus subtilis*) act in concert at a sequence designated *dif* ‘deletion-induced filamentation’ to resolve dimeric chromosomes after chromosome replication (Blakely *et al*., 1991, 1993; Sciochetti *et al*., 1999, 2001). The *dif* site is usually a 28-nucleotide motif associated with the chromosome's replication terminus and serves as template for chromosome dimer resolution. The sequence often contains palindromic motifs separated by a central hexanucleotide. In numerous bacteria, each side of the *dif* sequence is specifically targeted by one of the two Xer recombinases. An exception to this was documented for *Streptococci* and *Lactococci*, where an atypical 31-bp recombination *dif* site is recognized and processed by a single recombinase (Le Bourgeois *et al*., 2007).

A putative *dif* site (5′-GAAGGAA**ATAATGTATAT**G**ATG**G**TAAAT**-3′) was localized at position 230,387 (110° from the origin of replication) in *U. parvum* serovar 3 (ATCC 700970) (Yen *et al*., 2002) that shares high sequence identity to the *dif* sequence of *E. coli* (letters in bold). We have localized another potential *dif* site (*dif*_*UP*_: 5′-TGATATTTTAATGTATATTATTTATTCA-3′) in the *U. parvum* chromosome that is located 181° from the origin of replication (Fig. 1d). Alignment of these sequences with known *dif* sites from other bacteria showed high sequence identity in the central region (Fig. 1e).

In this publication, we took an approach to identify possible DNA-binding partners of the three potential *Ureaplasma* tyrosine recombinases. These DNA-binding partners were as follows: (i) the short inverted repeats of the ‘*mba* locus’ and the ‘UU172 phase-variable element’, (ii) the two potential *dif* sites, and (iii) the direct repeat flanking the 20-kb region. We demonstrate protein–DNA interaction for the three recombinases and discuss their possible functional roles.

## Materials and methods

### Southern blot

Genomic DNA from *U. parvum* serovar 3 cultures (strains ATCC 27815^T^, DR1, M14, V397, V890, V892) was isolated as described (Zimmerman *et al*., 2011) from 500 mL overnight cultures. DNA pellets were air-dried and re-suspended in 100 μL 1 × TE buffer for digestion with HincII. The digested DNA (20 μL per lane) was separated in a 1% agarose gel and transferred onto nylon membranes (Sambrook *et al*., 1989). Three DIG-11-dUTP-labeled PCR products were synthesized with recombinant Taq DNA polymerase for use as hybridization probes: UU145 (#145) with primers 5′-GCGGATCCATGGAGCGACAAAGTATG-3′ and 5′-CGAAGCTTATTTATCATTTTCAAATTC-3′, UU222 (#222) with primers 5′-GCGGATCCATGAAAGATTTTATTAGATA-3′ and 5′-CGAAGCTTATTCTGCATCATTTTGG-3′, and UU529 (#529) with primers 5′-GCGGATCCATGAAAAAATTTAT AAAT-3′ and 5′-CGAAGCTTAATTAACTTTTTTAT-3′. Hybridization and detection were carried out as described (Zimmerman *et al*., 2011). Hybridization was carried out in 5 × SSC/1% SDS at 53° C. In two separate blots, UU222 was detected prior to detection of either UU145 or UU529.

Genomic DNA from *Mycoplasma pneumoniae* M129 was isolated as described above from adhesive cells growing in 75-cm^2^ cell culture flasks and was re-suspended in 300–500 μL 1 × TE. Genomic DNA was digested with HindIII and BglII. Three DIG-11-dUTP-labeled PCR products were synthesized for use as hybridization probes: 400 bp of the 5′ region of the gentamicin resistance gene from plasmid pMT85 (Zimmerman & Herrmann, 2005) with primers 5′-GATGATGATTTTCCTTTGATG-3′ and 5′-ATGCCCTTATTGCTCTTGGAT-3′, the repeat region of the *mba* gene with primers 5′-ATTGGATCCACTACACAACCAGGT-3′ and 5′-TTATTTTCCAGTAGTTTCTTT-3′, and 322 bp of the 5′ region of UU376 with primers 5′-ATCTCCGACTCCAGCTCC-3′ and 5′-TTCATAGTCAACATT TGAAT-3′.

### Purification of recombinant proteins MBP::RipX, MBP::XerC, and MBP::CodV

Three recombinant proteins were expressed as fusions with the maltose-binding protein (MBP) of the expression vector pMAL-c2X (New England Biolabs) and purified by affinity chromatography over amylose. Gene *xerC* (UU222) was synthesized by ligating two PCR products and exchanging the internal TGA codon to TGG. A 6 × His-tag was added at the 3′ end of UU222. Genes *ripX* (UU145) and *codV* (UU529) were synthesized by Eurofins MWG Operon, with optimized codon usage for *E. coli* (accession # HF558294 and HF558295). Genes were fused between the restriction sites BamHI and HindIII of pMAL-c2X and constructs were cloned in *E. coli* DH10B (Invitrogen). Fusion proteins MBP::XerC and MBP::RipX were expressed from 400 mL broth cultures for 2 h with 0.5 mM IPTG. Fusion protein MBP::CodV was purified from 7 L broth culture. The soluble fractions of cell lysates were loaded onto 5 mL amylose, and fusion proteins were purified as described by the manufacturer (NEB; pMAL™ Protein Fusion and Purification System (Expression and Purification of Proteins and Cloned Genes) Instruction Manual, #E8000S Version 5.3 11/07, Affinity Chromatography, Method I).

### Electrophoretic mobility shift assay

Electrophoretic mobility shift assay (EMSA) analysis was carried out with the LightShift® Chemoluminescent EMSA Kit (PIERCE) according to the product manual. Reactions were carried out in a final volume of 20 μL at 20° C for 20 min, prior to loading onto a polyacrylamide gel in 0.5 × TBE buffer. Labeled DNA always had a concentration of 10 fmol per reaction. The protein concentration was 500 ng (ca. 490 nM for MBP and 340 nM for fusion proteins) per reaction, and the MgCl_2_ concentration was 7.5 mM, unless otherwise specified.

A 145-bp PCR product with the 24-bp IR_*mba*_ located between positions 87 and 111 was synthesized from the *mba* locus with biotinylated primers 5′-ATCGATAACATTATTAGATAT-3′ and 5′-TTGTTGGCTTGGAGCTGAAG-3′.

Short double-stranded DNA was generated by annealing oligonucleotides in 10 mM Tris/HCl pH 7.5, 100 mM NaCl, and 1 mM EDTA during a temperature gradient from 85° C to 25° C. The following biotinylated probes (Fig. S2) were constructed: IR_*mba*_ (5′-TTCAAAGTTCACTTTTTCT GTTTGATAATTCAAAT-3′), IR_UU172_ (5′-TTAAATAATGATA ATTAAATTATCAAACAGTAACTTTT-3′), *dif*_*UP*_ (5′-ATGATA TTTTAATGTATATTATTTATTCAT-3′), *dif*_Yen_ (5′-TGAAGG AAATAATGTATATGATGGTAAATC-3′), and DR_20-kb_ (5′-AA CAAGGTTCAATAATCACGATTATTAAA-3′). Two non-biotinylated competitor DNA probes were generated: IR_*mba*_ (5′-T TTCTGTTTGATAATTCAAATTA-3′) and IR_UU172_ (5′-TAAA TTATCAAACAGTAACTTTT-3′).

### Construction of vectors for transformation of *M. pneumoniae*

#### Construction of pMT::mba^trunc^

An ‘*mba* locus’ was constructed by ligating two PCR products together, exchanging the TGA codon in the 5′ region of the *mba* gene to TGG and adding a HindIII restriction site 3′ to the stop codon of the *mba* gene. This *mba* locus was digested with the restriction endonucleases HindIII and HpaI, truncating the UU376 gene at the 3′ end by 21 nucleotides (six amino acids) with HpaI to eliminate the third IR_*mba*_ found in the intergenic region 3′ of UU376. This truncated locus (*mba*^trunc^) was ligated between the BstZ17I and HindIII sites of a modified Tn*4001* vector plasmid pMT85 (Zimmerman & Herrmann, 2005) that contains a resistance cassette against gentamicin, yielding pMT::*mba*^trunc^ (Fig. S3). *Mycoplasma pneumoniae* M129 was transformed with pMT::*mba*^trunc^ by electroporation (Hedreyda *et al*., 1993), and a clone (MP*mba*^trunc^) with single genomic integration at position 495,321 at the 3′ end of the hypothetical gene MPN411 was chosen for further experiments.

#### Construction of pCT::UU222

Gene UU222 was PCR amplified from genomic DNA of *U. parvum* serovar 3 and ligated with a 275-bp upstream region of UU529 (529P) that served as active promoter, using an NdeI site as linker between promoter and gene. Activity of the putative promoter region in *M. pneumoniae* was first tested by linking the 275 bp to the gene *mrfp1* (Campbell *et al*., 2002) in pMT85 (Zimmerman & Herrmann, 2005) and following Mrfp1 (monomeric red fluorescent protein) expression by Western blot from subclones (Fig. S4). The 529P::UU222 construct was ligated between the BamHI and EcoRI sites of the modified Tn*4001* vector plasmid pCT461 (Herrmann, unpublished) that contains a resistance cassette against chloramphenicol, yielding pCT::UU222. Clone MP*mba*^trunc^ was transformed with pCT::UU222 as described above.

## Results

### Screening of recombinase genes in *U. parvum* serovar 3 strains

**Fig. 2:**
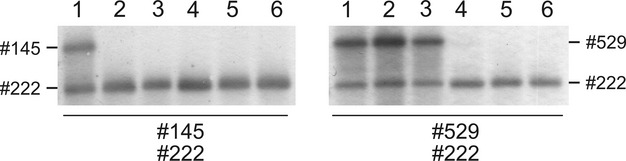
Southern blot detection of genes: *ripX*, *xerC,* and *codV*. Detection of UU145, UU222, and UU529 in different *Ureaplasma parvum* serovar 3 strains (lanes 1–6: ATCC27815^T^, DR-1, M14, V397, V890, V892) that showed phase variation in the ‘*mba* locus’ and the ‘UU172 phase-variable element’. Chromosomal DNA was digested with HincII, separated in a 1% agarose gel, and transferred onto nylon membranes. Expected fragment sizes: 3193 bp for #222, 6890 bp for #145, and 6201 bp for #529. Bands were detected with Dig-11-dUTP-labeled PCR probes (#145, #222, #529) comprising the entire sequences of the genes UU145, UU222, and UU529.

Three recombinases belonging to the tyrosine family have been annotated in *U. parvum* serovar 3 (ATCC 700970) (Glass *et al*., 2000). In this strain, the genes received the locus tags UU145, UU222, and UU529 for *ripX*, *xerC,* and *codV*, respectively. Of the three potential tyrosine recombinases, only orthologs of *xerC* have been annotated in all 14 *Ureaplasma* serovars (Paralanov *et al*., 2012); *ripX* seems to be absent in several strains, while *codV* has been annotated only in *U. parvum* strains (Table S1). Momynaliev *et al*. (2007) likewise documented the absence of *ripX* and *codV* in several *U. parvum* strains. We carried out Southern blot analyses with genomic DNA from five clinical *U. parvum* serovar 3 strains and the sequenced type strain ATCC 27815^T^ and screened for the presence of the three recombinase-encoding genes with gene-specific probes. Results indicated that only orthologs of *xerC* (UU222) are present in all six strains (Fig. 2); UU529 was detected in three strains, while UU145 was found present only in the type strain.

### *In vitro* binding of fusion proteins to DNA substrates

All three putative recombinase genes were cloned and expressed in *E. coli*. After removal of internal TGA codons, genes were cloned into the expression vector pMAL-c2X as fusions with the MBP encoding gene. This system was chosen, as His-tagged fusions of XerC proved to be highly insoluble (data not shown). Expression of MBP::CodV was meager and required greater amounts of cells for a higher protein yield. We attribute this to the lethal properties of CodV to *E. coli* as the cell titer dropped upon induction and protein expression was low (Fig. S5 and S6). Moreover, DAPI staining of DNA from induced cells indicated DNA degradation (Fig. S7). The soluble protein fraction of *E. coli* DH10B and the purified MBP alone were used as controls. Expressed proteins were purified by affinity chromatography, observed by SDS-PAGE (Fig. S8), and used in EMSA experiments.

**Fig. 3:**
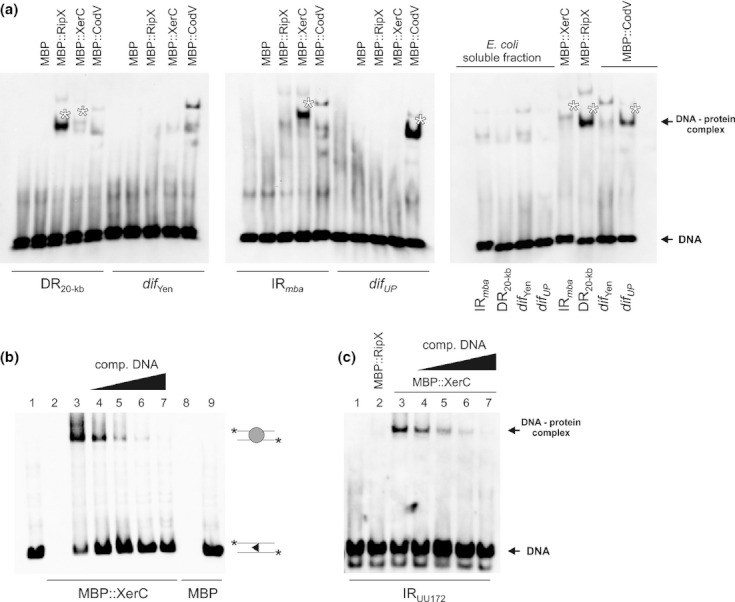
Binding of RipX, XerC, and CodV to substrate DNA. (a) Protein–DNA interaction of purified proteins and the soluble protein fraction of *Escherichia coli* DH10B with different biotinylated substrate DNAs. For each reaction, 250 ng of protein was used (except MBA::CodV, where 400 ng was used). Specific interactions of recombinant fusion proteins with substrate DNA are labeled with an asterisk. (b) Specific binding of XerC to IR_*mba*_. EMSA analysis using a purified MBP::XerC fusion (•) and a biotinylated (*) PCR product of 145 bp containing one inverted repeat (◄) (IR_*mba*_). Lane 1, PCR; lane 2, MBP::XerC; lane 3, PCR + MBP::XerC; lanes 4–7, PCR + MBP::XerC + increasing concentrations of a short 23-bp IR_*mba*_ competitor DNA (1, 3, 10, and 30 pmol); lane 8, MBP; lane 9, PCR + MBP. (c) Binding of XerC to IR_UU__172_. EMSA analysis using purified MBP::XerC or MBP::RipX and the biotinylated inverted repeat IR_UU__172_. Lane 1, IR_UU__172_; lane 2, IR_UU__172_ + MBP::RipX; lane 3, IR_UU__172_ + MBP::XerC; lanes 4–7, IR_UU__172_ + MBP::XerC + increasing concentrations of the IR_UU__172_ competitor DNA (1, 3, 10, and 30 pmol).

Electrophoretic mobility shift assay analyses with the purified proteins and the annealed templates indicated a binding specificity of XerC for the inverted repeat IR_*mba*_, of RipX for the direct repeat DR_20-kb_, and of CodV for the potential *dif*_*UP*_ site (Fig. 3a). Interaction of XerC with DR_20-kb_ was also observed (left panel) and the signal enhanced with increased protein concentration (Fig. S9). MBP alone did not bind to the DNA substrates. A further protein–DNA complex was observed with probes MBP::CodV and *dif*_Yen_; however, this band ran above the expected height and is attributable to binding of background *E. coli* proteins in the protein preparation. This false-positive band can be observed in reactions using the soluble protein fraction of *E. coli* with the same substrate DNA (Fig. 3a, right panel, and Fig. S10).

To enhance the signal and to test whether binding of XerC to IR_*mba*_ was specific, we synthesized a 145-bp-long PCR product from the ‘*mba* locus’ that contained one inverted repeat and applied it in competition analysis using a short 23-bp IR_*mba*_ as competitor DNA. Binding of XerC proved to be specific for the IR_*mba*_ sequence (Fig. 3b). Similar results were obtained with XerC and IR_UU172_, using a short 23-bp competitor DNA (Fig. 3c).

**Fig. 4:**
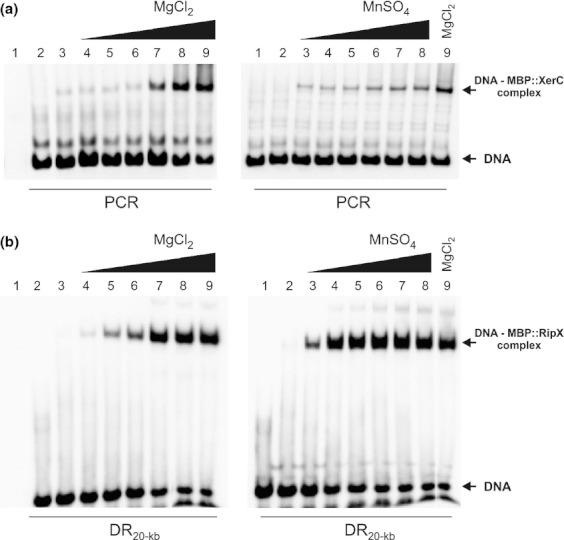
Divalent cation-dependent protein–DNA interaction. Magnesium- and manganese-dependent binding of MBP::XerC and MBP::RipX to substrate DNA *in vitro*. (a) Left panel: EMSA analysis using a purified MBP fusion of XerC and a biotinylated PCR product of 145 bp containing one inverted repeat IR_*mba*_. Lane 1, MBP::XerC; lane 2, PCR; lane 3, PCR + MBP::XerC; lanes 4–9, PCR + MBP::XerC, and increasing MgCl_2_ concentration (0.5, 0.75, 1, 2.5, 5, and 7.5 mM) in the binding reaction. Right panel: EMSA analysis using a purified MBP fusion of XerC and a biotin-labeled PCR product. Lane 1, PCR; lane 2, PCR + MBP::XerC; lanes 3–8, PCR + MBP::XerC and increasing MnSO_4_ concentration (0.5, 0.75, 1, 2.5, 5, and 7.5 mM) in the binding reaction; lane 9, PCR + MBP::XerC and 7.5 mM MgCl_2_ in the binding reaction. (b) Left panel: EMSA analysis using a purified MBP fusion of RipX and the biotinylated substrate DR_20-kb_. Lane 1, MBP::RipX; lane 2, DR_20-kb_; lane 3, DR_20-kb_ + MBP::RipX; lanes 4–9, DR_20-kb_ + MBP::RipX and increasing MgCl_2_ concentration (0.5, 0.75, 1, 2.5, 5, and 7.5 mM) in the binding reaction. Right panel: Lane 1, DR_20-kb_; lane 2, DR_20-kb_ + MBP::RipX; lanes 3–8, DR_20-kb_ + MBP::RipX and increasing MnSO_4_ concentration (0.5, 0.75, 1, 2.5, 5, and 7.5 mM) in the binding reaction; lane 9, DR_20-kb_ + MBP::RipX and 7.5 mM MgCl_2_ in the binding reaction.

EDTA inhibited protein–DNA interaction (Fig. S11). We therefore tested whether binding of XerC and RipX to their DNA substrates was cation dependent. Interactions of MBP::XerC with IR_*mba*_ and MBP::RipX with DR_20-kb_ could be enhanced with either MgCl_2_ or MnSO_4_, indicating divalent cation-dependent binding (Fig. 4).

### XerC-mediated inversion of the mba locus

**Fig. 5:**
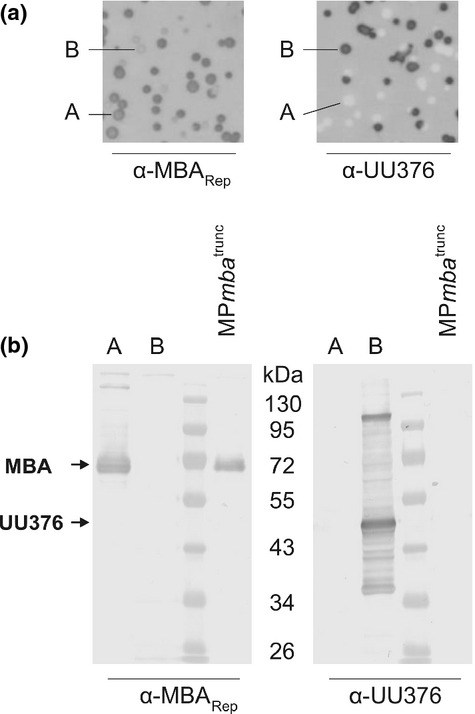
MBA and UU376 expression in clonal variants of MP*mba*^trunc^. (a) Colony immunoblot of MP*mba*^trunc^ that had been transformed with pCT::UU222, grown in medium containing 80 μg mL^−1^ gentamicin and 25 μg mL^−1^ chloramphenicol, plated on agar, and transferred onto nitrocellulose membrane. Protein expression of MBA and UU376 was detected with mono-specific antibodies against the repetitive region of the MBA protein (α-MBA_Rep_) or the UU376 protein (α-UU376) (Zimmerman *et al*., 2009) and made visible with a horse radish peroxidase–conjugated secondary antibody. (b) Western blot analysis with total protein from clone MP*mba*^trunc^ and from clones of variant A and B. Immunostaining was carried out as in (a). Variants A and B showed phase-locked expression of MBA and UU376, respectively.

**Fig. 6:**
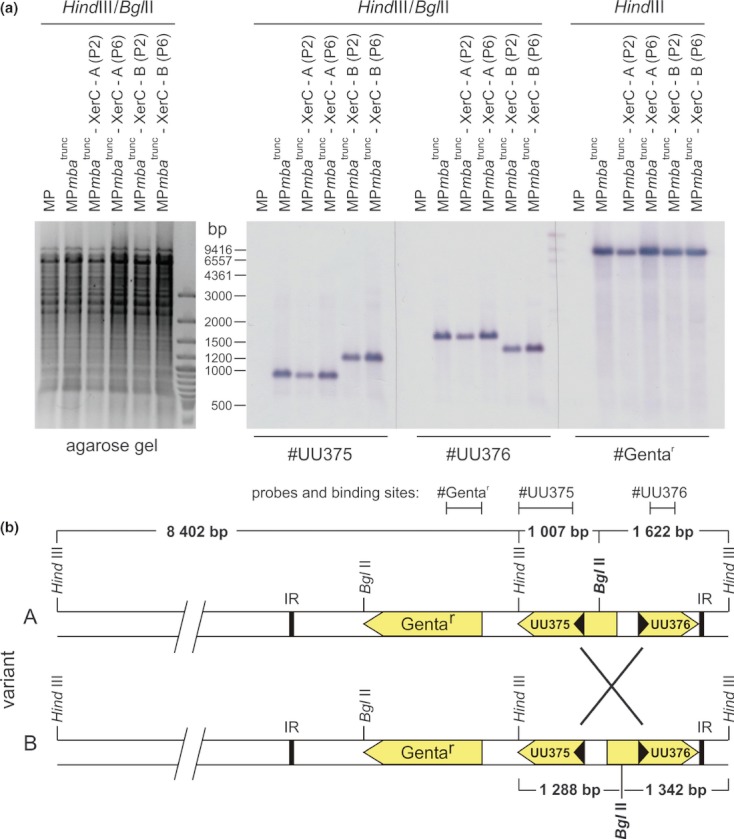
DNA inversion in the ‘*mba* locus’. (a) Southern blot analysis with genomic DNA of *Mycoplasma pneumoniae* M129 (MP), MP that was transformed with plasmid pMT::*mba*^trunc^ (MP*mba*^trunc^), and two clonal variants (A and B) of MP*mba*^trunc^ that had been isolated after transformation with plasmid pCT::UU222 (indicated by XerC). Genomic DNA of variants A and B was isolated after the second and sixth growth passage (P2 and P6). DNA was digested with HindIII and BglII and hybridized with the DIG-11-dUTP-labeled probes #Genta^r^, #UU375, and #UU376. Probe #Genta^r^ was used for determining single integration of the insert and detected an 8402-bp fragment in HindIII-digested DNA. Probes #UU375 and #UU376 were used for detecting *mba* locus configuration and DNA inversion within the *mba* locus before and after co-transformation of MP*mba*^trunc^ with pCT::UU222. Probe #UU375 detected a 1007-bp fragment in the unaltered *mba* locus of BglII/HindIII-digested DNA and a 1288-bp fragment in the locus that had undergone DNA inversion. Similarly, probe #UU376 detected a 1622-bp fragment in the unaltered *mba* locus of BglII/HindIII-digested DNA and a 1342-bp fragment in the locus that had undergone DNA inversion. (b) Schematic illustration of the DNA inversion event in the ‘*mba* locus’ that had been integrated in the genome of *M. pneumoniae*. Integration of the *mba* locus (*mba*^trunc^) had occurred at chromosome position 495,321 via the inverted repeats (IR) of the insertion element located in plasmid pMT::*mba*^trunc^ with concurrent elimination of the transposase gene (see Fig. S12). The *mba* locus of variant A corresponds to that of MP*mba*^trunc^, while that of variant B has undergone DNA inversion. Captions and labeling: Genta^r^, gentamicin resistance gene; black triangle, short inverted repeat; and black cross, DNA inversion.

The EMSA results suggested XerC as potential mediator in the DNA inversion event associated with MBA phase variation. To test whether DNA inversion is mediated by XerC, the model organism *M. pneumoniae* was co-transformed with two plasmids, one carrying a truncated ‘*mba* locus’ with two IR_*mba*_ sequences and the other harboring the recombinase gene *xerC* fused behind an active promoter. An *M. pneumoniae* clone (MP*mba*^trunc^) with the *mba* locus integrated at the genomic position 495,321 was first generated by transforming *M. pneumoniae* M129 with plasmid pMT::*mba*^trunc^. MBA and UU376 protein expression in MP*mba*^trunc^ was screened by Western blot and colony blot throughout eight passages, showing no alternating expression (data not shown); that is, only MBA and no UU376 protein was expressed at all times in subclones. Clone MP*mba*^trunc^ from the fourth passage was transformed with pCT::UU222 and subcloned. Although the *xerC* had not been integrated into the genome, subclones of transformed MP*mba*^trunc^ now showed either MBA (variant A) or UU376 (variant B) expression (Fig. 5). Southern blot analysis with genomic DNA showed that DNA inversion had taken place in variant B (Fig. 6). We repeated the transformation experiment, however, obtained the same result; that is, subclones showed phase-locked expression for either MBA or UU376, but did not have the desired integration of *xerC* into the genome.

## Discussion

We have identified binding sites of the three potential tyrosine recombinases of *U. parvum* serovar 3. XerC was found to interact with the short inverted repeats located within the two phase-variable gene clusters that have been described as the ‘*mba* locus’ and the ‘UU172 phase-variable element’, suggesting its involvement in promoting the postulated site-specific recombination event that leads to antigenic variation of major surface proteins. DNA inversion was observed within the ‘*mba* locus’ after co-transformation of *M. pneumoniae* with both the ‘*mba* locus’ and the *xerC* gene located behind an active promoter. Unfortunately, we were unable to follow alternating expression of MBA and UU376 in *M. pneumoniae*, as the recombinase gene had not integrated into the organism's genome. We believe that the active XerC protein in the transformed clone MP*mba*^trunc^ processed DNA inversion of the ‘*mba* locus’ before the vector was degraded, and subclones were phase-locked for either MBA or UU376 expression. Chloramphenicol resistance is frequently acquired by *M. pneumoniae* after electroporation, which explains the antibiotic resistance of the false-positive subclones.

The fact that only *xerC* is present in some *Ureaplasma* strains that showed high-frequency phase variation in the two loci supports the idea that only one tyrosine recombinase is involved in the site-specific recombination event of these loci. Recombination mechanisms in mycoplasmas, where only a single recombinase mediates site-specific recombination, have been described for the *hsd* and *vsr* systems of *M. pulmonis* (Sitaraman *et al*., 2002), the *mpl* system of *M. penetrans* (Horino *et al*., 2009), and the *vpma* system of *M. agalactiae* (Czurda *et al*., 2010). Because all analyzed *Ureaplasma* strains showed high-frequency phase variation in both the ‘*mba* locus’ and the ‘UU172 phase-variable element’ in our previous studies, the absence of UU145 and UU529 suggests that their encoded proteins are neither required for site-specific recombination in these phase-variable loci nor essential for *in vivo* or *in vitro* growth.

The core-binding domain of *Ureaplasma* RipX has previously been aligned with other integrases (Swalla *et al*., 2003). This, and the proposal of a putative *dif* site within the *Ureaplasma* genome (Yen *et al*., 2002), prompted us to investigate possible binding of the potential tyrosine recombinases to this site. Interestingly, none of the tested fusion proteins bound to the proposed *dif* site. However, CodV was found to interact with another potential *dif* site that is located 181° away from the origin of replication. The finding suggests an involvement of *Ureaplasma* CodV in a chromosome dimer resolution event. The fact that *codV* is not present in all *Ureaplasma* strains, however, indicates that the proposed event might be processed by other enzymes, such as the translocase FtsK and the topoisomerase IV complex, whose genes are present in all sequenced *Ureaplasma* genomes (Table S1). It could likewise be that the gene *codV* encodes an unessential protein of the chromosome dimer resolution mechanism, left over after genome reduction in some strains, or has been acquired by horizontal gene transfer, or is responsible for yet another unknown mechanism. Horizontal gene transfer has recently been described to occur among ureaplasmas, but also with other *Mycoplasma* species (Pereyre *et al*., 2009; Xiao *et al*., 2011; Paralanov *et al*., 2012), and has been suggested for the occurrence of *ripX* which, like *codV*, is present only in a subset of isolates. Interestingly, orthologs of *codV* have so far only been annotated for the *U. parvum* species and seem to be missing in *U. urealyticum*.

Our results suggest that XerC of *U. parvum* serovar 3 is a mediator in the proposed DNA inversion event of the two phase-variable loci. We postulate that RipX is a potential mediator in the integration of a mobile element. Further analyses focusing on the recombination mechanisms are needed to elucidate the direct functional roles of these potential enzymes in the proposed recombination events.

## Supporting Information

**Fig. S1.** Alignment of three putative tyrosine recombinases of *Ureaplasma parvum* serovar 3 (ATCC 700970 and ATCC 27815^T^).

**Fig. S2.** Annealed oligonucleotides for EMSA analyses.

**Fig. S3.** Plasmid pMT::*mba*^trunc^.

**Fig. S4.** Promoter fusion and Mrfp1 expression from *Ureaplasma* promoters in *Mycoplasma pneumoniae* M129.

**Fig. S5.** Growth curve of MBP::CodV expressing *Escherichia coli*.

**Fig. S6.** Protein expression and purification of MBP fusion proteins.

**Fig. S7.** Cell morphology of MBP::CodV expressing *Escherichia coli*.

**Fig. S8.** Protein preparations for EMSA analyses.

**Fig. S9.** Protein concentration-dependent binding of MBP::RipX and MBP::XerC to DR_20-kb_.

**Fig. S10.** Protein-DNA interaction.

**Fig. S11.** Inhibition of XerC binding to IR_*mba*_ by EDTA.

**Fig. S12.** Integration of *mba*^trunc^ into the *Mycoplasma pneumoniae* chromosome.

**Fig. S13.** Protein concentration-dependent binding of MBP::RipX and MBP::XerC to IR_*mba*_.

**Fig. S14.** Protein concentration-dependent binding of MBP::XerC to IR_*mba*_ on a 145-bp PCR product.

**Fig. S15.** Protein concentration-dependent binding of MBP::CodV and the soluble fraction of *Escherichia coli* DH10B to *dif*_*UP*_.

**Table S1.** Occurrence of the three putative tyrosine recombinase-encoding genes *ripX*, *xerC* and *codV* in *Ureaplasma* serovars, the translocase encoding gene *ftsK* and genes encoding topoisomerase subunits *parE* and *parC*.

**Table S2.** Growth of MBP::CodV expressing *Escherichia coli*.

**Table S3.** Features of proteins used for EMSA analyses.
