# Supplementary material for: Interaction of the putative tyrosine recombinases RipX (UU145), XerC (UU222), and CodV (UU529) of Ureaplasma parvum serovar 3 with specific DNA
Source: FEMS Microbiol Lett. 2013 Jan 31;340(1):55–64. doi: 10.1111/1574-6968.12077 (PMC3599477; doi:10.1111/1574-6968.12077)
Supplement: Supplementary file 1 [file fml0340-0055-SD1.doc]

**Table S1. Occurrence of the three putative tyrosine recombinase-encoding genes *ripX*, *xerC* and *codV* in *Ureaplasma* serovars, the translocase encoding gene *ftsK* and genes encoding topoisomerase subunits *parE* and *parC* .**

| **Ser** | **Strain** | **UU145 (*ripX*)*** | **UU222 (x*erC*)*** | **UU529 (*codV*)*** | **UU508(*ftsK*)*** | **UU081 (*parE*)** | **UU082 (*parC*)** | **UU466 (*parE*)** | **UU467 (*parC*)** |
| --- | --- | --- | --- | --- | --- | --- | --- | --- | --- |
| 3 | ATCC 700970 | [NP_077976.1](http://www.ncbi.nlm.nih.gov/protein/13357702) | [NP_078055.1](http://www.ncbi.nlm.nih.gov/protein/13357781) | [NP_078367.1](http://www.ncbi.nlm.nih.gov/protein/13358093) | [NP_078345.1](http://www.ncbi.nlm.nih.gov/protein/13358071) | [NP_077911.1](http://www.ncbi.nlm.nih.gov/protein/13357637?report=genbank&log$=protalign&blast_rank=1&RID=1ZCJH80W01S) | [NP_077912.1](http://www.ncbi.nlm.nih.gov/protein/13357638?report=genbank&log$=protalign&blast_rank=1&RID=1ZDT6SU301N) | [NP_078303.1](http://www.ncbi.nlm.nih.gov/protein/13358029?report=genbank&log$=protalign&blast_rank=1&RID=1ZGVTRBM012) | [NP_078304.1](http://www.ncbi.nlm.nih.gov/protein/13358030?report=genbank&log$=protalign&blast_rank=1&RID=1ZJW93WT01S) |
| 3 | ATCC 27815^T^ | [YP_001752228.1](http://www.ncbi.nlm.nih.gov/protein/170762035?report=genbank&log$=protalign&blast_rank=2&RID=NRP82ZMJ011) | [YP_001752304.1](http://www.ncbi.nlm.nih.gov/protein/170762132?report=genbank&log$=protalign&blast_rank=1&RID=NRMMTAJ2011) | [YP_001752614.1](http://www.ncbi.nlm.nih.gov/protein/170762050?report=genbank&log$=protalign&blast_rank=1&RID=NRZ757ZJ011) | [YP_001752592.1](http://www.ncbi.nlm.nih.gov/protein/170762039?report=genbank&log$=protalign&blast_rank=1&RID=PC8KDU80011) | [YP_001752160.1](http://www.ncbi.nlm.nih.gov/protein/170762124?report=genbank&log$=protalign&blast_rank=1&RID=1ZCJH80W01S) | [YP_001752161.1](http://www.ncbi.nlm.nih.gov/protein/170761893?report=genbank&log$=protalign&blast_rank=1&RID=1ZDT6SU301N) | [YP_001752551.1](http://www.ncbi.nlm.nih.gov/protein/170761866?report=genbank&log$=protalign&blast_rank=1&RID=1ZGVTRBM012) | [YP_001752552.1](http://www.ncbi.nlm.nih.gov/protein/170761865?report=genbank&log$=protalign&blast_rank=1&RID=1ZJW93WT01S) |
| 3 | DR1 | - | + | + | ND | ND | ND | ND | ND |
| 3 | M14 | - | + | + | ND | ND | ND | ND | ND |
| 3 | V397 | - | + | - | ND | ND | ND | ND | ND |
| 3 | V890 | - | + | - | ND | ND | ND | ND | ND |
| 3 | V892 | - | + | - | ND | ND | ND | ND | ND |
| 1 | ATCC 27813 | -- | [ZP_02931743.1](http://www.ncbi.nlm.nih.gov/protein/171920428?report=genbank&log$=protalign&blast_rank=1&RID=NRMMTAJ2011) | [ZP_02931574.1](http://www.ncbi.nlm.nih.gov/protein/171920189?report=genbank&log$=protalign&blast_rank=1&RID=NRZ757ZJ011) | [ZP_02931551.1](http://www.ncbi.nlm.nih.gov/protein/171920151?report=genbank&log$=protalign&blast_rank=1&RID=PC8KDU80011) | [ZP_02931785.1](http://www.ncbi.nlm.nih.gov/protein/171920490?report=genbank&log$=protalign&blast_rank=1&RID=1ZCJH80W01S) | [ZP_02931663.1](http://www.ncbi.nlm.nih.gov/protein/171920319?report=genbank&log$=protalign&blast_rank=1&RID=1ZDT6SU301N) | [ZP_02931723.1](http://www.ncbi.nlm.nih.gov/protein/171920400?report=genbank&log$=protalign&blast_rank=4&RID=1ZGVTRBM012) | [ZP_02931772.1](http://www.ncbi.nlm.nih.gov/protein/171920470?report=genbank&log$=protalign&blast_rank=4&RID=1ZJW93WT01S) |
| 2 | ATCC 27814 | -- | [ZP_03771707.1](http://www.ncbi.nlm.nih.gov/protein/225550758?report=genbank&log$=protalign&blast_rank=1&RID=NUB1320K01S) | -- | [ZP_03771283.1](http://www.ncbi.nlm.nih.gov/protein/225550334?report=genbank&log$=protalign&blast_rank=3&RID=PC8KDU80011) | [ZP_03771719.1](http://www.ncbi.nlm.nih.gov/protein/225550770?report=genbank&log$=protalign&blast_rank=2&RID=1ZCJH80W01S) | [ZP_03771864.1](http://www.ncbi.nlm.nih.gov/protein/225550915?report=genbank&log$=protalign&blast_rank=3&RID=1ZDT6SU301N) | [ZP_03771636.1](http://www.ncbi.nlm.nih.gov/protein/225550687?report=genbank&log$=protalign&blast_rank=5&RID=1ZGVTRBM012) | [ZP_03771729.1](http://www.ncbi.nlm.nih.gov/protein/225550780?report=genbank&log$=protalign&blast_rank=10&RID=1ZJW93WT01S) |
| 4 | ATCC 27816 | [ZP_03206315.1](http://www.ncbi.nlm.nih.gov/protein/198273781?report=genbank&log$=protalign&blast_rank=3&RID=NRP21EN5014) | [ZP_03206102.1](http://www.ncbi.nlm.nih.gov/protein/198273566?report=genbank&log$=protalign&blast_rank=1&RID=NRN2WWZ5016) | -- | [ZP_03205815.1](http://www.ncbi.nlm.nih.gov/protein/198273279?report=genbank&log$=protalign&blast_rank=2&RID=PC8KDU80011) | [ZP_03206098.1](http://www.ncbi.nlm.nih.gov/protein/198273562?report=genbank&log$=protalign&blast_rank=2&RID=1ZCJH80W01S) | [ZP_03205980.1](http://www.ncbi.nlm.nih.gov/protein/198273444?report=genbank&log$=protalign&blast_rank=2&RID=1ZDT6SU301N) | [ZP_03206311.1](http://www.ncbi.nlm.nih.gov/protein/198273777?report=genbank&log$=protalign&blast_rank=6&RID=1ZGVTRBM012) | [ZP_03206351.1](http://www.ncbi.nlm.nih.gov/protein/198273817?report=genbank&log$=protalign&blast_rank=7&RID=1ZJW93WT01S) |
| 5 | ATCC 27817 | [ZP_02964732.1](http://www.ncbi.nlm.nih.gov/protein/185178975?report=genbank&log$=protalign&blast_rank=1&RID=NRP21EN5014) | [ZP_02554882.2](http://www.ncbi.nlm.nih.gov/protein/185178831?report=genbank&log$=protalign&blast_rank=2&RID=NRMMTAJ2011) | -- | [ZP_02964768.1](http://www.ncbi.nlm.nih.gov/protein/185179018?report=genbank&log$=protalign&blast_rank=2&RID=PC8KDU80011) | [ZP_02554678.2](http://www.ncbi.nlm.nih.gov/protein/185178763?report=genbank&log$=protalign&blast_rank=2&RID=1ZCJH80W01S) | [ZP_02964532.1](http://www.ncbi.nlm.nih.gov/protein/185178715?report=genbank&log$=protalign&blast_rank=2&RID=1ZDT6SU301N) | [ZP_02964846.1](http://www.ncbi.nlm.nih.gov/protein/185179126?report=genbank&log$=protalign&blast_rank=5&RID=1ZGVTRBM012) | [ZP_02964849.1](http://www.ncbi.nlm.nih.gov/protein/185179130?report=genbank&log$=protalign&blast_rank=8&RID=1ZJW93WT01S) |
| 6 | ATCC 27818 | [ZP_02971457.1](http://www.ncbi.nlm.nih.gov/protein/186701779?report=genbank&log$=protalign&blast_rank=3&RID=NRP82ZMJ011) | [ZP_02971618.1](http://www.ncbi.nlm.nih.gov/protein/186701999?report=genbank&log$=protalign&blast_rank=1&RID=NRMMTAJ2011) | [ZP_02971333.1](http://www.ncbi.nlm.nih.gov/protein/186701629?report=genbank&log$=protalign&blast_rank=1&RID=NRZ757ZJ011) | [ZP_02971524.1](http://www.ncbi.nlm.nih.gov/protein/186701864?report=genbank&log$=protalign&blast_rank=1&RID=PC8KDU80011) | [ZP_02553290.2](http://www.ncbi.nlm.nih.gov/protein/186701878?report=genbank&log$=protalign&blast_rank=1&RID=1ZCJH80W01S) | [ZP_02971542.1](http://www.ncbi.nlm.nih.gov/protein/186701885?report=genbank&log$=protalign&blast_rank=1&RID=1ZDT6SU301N) | [ZP_02971474.1](http://www.ncbi.nlm.nih.gov/protein/186701799?report=genbank&log$=protalign&blast_rank=2&RID=1ZGVTRBM012) | [ZP_02971413.1](http://www.ncbi.nlm.nih.gov/protein/186701721?report=genbank&log$=protalign&blast_rank=2&RID=1ZJW93WT01S) |
| 7 | ATCC 27819 | [ZP_02997125.1](http://www.ncbi.nlm.nih.gov/protein/188024479?report=genbank&log$=protalign&blast_rank=2&RID=NRP21EN5014) | [ZP_02570121.2](http://www.ncbi.nlm.nih.gov/protein/188024209?report=genbank&log$=protalign&blast_rank=2&RID=NRMMTAJ2011) | -- | [ZP_02996814.1](http://www.ncbi.nlm.nih.gov/protein/188024069?report=genbank&log$=protalign&blast_rank=5&RID=PC8KDU80011) | [ZP_02570760.2](http://www.ncbi.nlm.nih.gov/protein/188024457?report=genbank&log$=protalign&blast_rank=2&RID=1ZCJH80W01S) | [ZP_02997078.1](http://www.ncbi.nlm.nih.gov/protein/188024419?report=genbank&log$=protalign&blast_rank=2&RID=1ZDT6SU301N) | [ZP_02996806.1](http://www.ncbi.nlm.nih.gov/protein/188024055?report=genbank&log$=protalign&blast_rank=5&RID=1ZGVTRBM012) | [ZP_02996816.1](http://www.ncbi.nlm.nih.gov/protein/188024071?report=genbank&log$=protalign&blast_rank=5&RID=1ZJW93WT01S)  [ZP_02996867.1](http://www.ncbi.nlm.nih.gov/protein/188024134?report=genbank&log$=protalign&blast_rank=6&RID=1ZJW93WT01S) |
| 8 | ATCC 28618 | [ZP_03772570.1](http://www.ncbi.nlm.nih.gov/protein/225551624?report=genbank&log$=protalign&blast_rank=3&RID=NRP21EN5014) | [ZP_03771989.1](http://www.ncbi.nlm.nih.gov/protein/225551043?report=genbank&log$=protalign&blast_rank=1&RID=NRN2WWZ5016) | -- | [ZP_03772218.1](http://www.ncbi.nlm.nih.gov/protein/225551272?report=genbank&log$=protalign&blast_rank=2&RID=PC8KDU80011) | [ZP_03772108.1](http://www.ncbi.nlm.nih.gov/protein/225551162?report=genbank&log$=protalign&blast_rank=2&RID=1ZCJH80W01S) | [ZP_03771985.1](http://www.ncbi.nlm.nih.gov/protein/225551039?report=genbank&log$=protalign&blast_rank=2&RID=1ZDT6SU301N) | [ZP_03772014.1](http://www.ncbi.nlm.nih.gov/protein/225551068?report=genbank&log$=protalign&blast_rank=5&RID=1ZGVTRBM012) | [ZP_03772352.1](http://www.ncbi.nlm.nih.gov/protein/225551406?report=genbank&log$=protalign&blast_rank=5&RID=1ZJW93WT01S) |
| 9 | ATCC 33175 | [ZP_03079860.1](http://www.ncbi.nlm.nih.gov/protein/195867861?report=genbank&log$=protalign&blast_rank=4&RID=NRP21EN5014) | [ZP_03079767.1](http://www.ncbi.nlm.nih.gov/protein/195867766?report=genbank&log$=protalign&blast_rank=1&RID=NRN2WWZ5016) | -- | [ZP_03079937.1](http://www.ncbi.nlm.nih.gov/protein/195867939?report=genbank&log$=protalign&blast_rank=2&RID=PC8KDU80011) | [ZP_03079485.1](http://www.ncbi.nlm.nih.gov/protein/195867481?report=genbank&log$=protalign&blast_rank=2&RID=1ZCJH80W01S) | [ZP_03079464.1](http://www.ncbi.nlm.nih.gov/protein/195867460?report=genbank&log$=protalign&blast_rank=2&RID=1ZDT6SU301N) | -- | [ZP_03079813.1](http://www.ncbi.nlm.nih.gov/protein/195867813?report=genbank&log$=protalign&blast_rank=5&RID=1ZJW93WT01S) |
| 10 | ATCC 33699 | [YP_002284801.1](http://www.ncbi.nlm.nih.gov/protein/209554523?report=genbank&log$=protalign&blast_rank=3&RID=NRP21EN5014) | [YP_002284620.1](http://www.ncbi.nlm.nih.gov/protein/209554447?report=genbank&log$=protalign&blast_rank=1&RID=NRN2WWZ5016) | -- | [YP_002284955.1](http://www.ncbi.nlm.nih.gov/protein/209554061?report=genbank&log$=protalign&blast_rank=2&RID=PC8KDU80011) | [YP_002284500.1](http://www.ncbi.nlm.nih.gov/protein/209554430?report=genbank&log$=protalign&blast_rank=2&RID=1ZCJH80W01S) | [YP_002284501.1](http://www.ncbi.nlm.nih.gov/protein/209554572?report=genbank&log$=protalign&blast_rank=3&RID=1ZDT6SU301N) | [YP_002284909.1](http://www.ncbi.nlm.nih.gov/protein/209554526?report=genbank&log$=protalign&blast_rank=6&RID=1ZGVTRBM012) | [YP_002284910.1](http://www.ncbi.nlm.nih.gov/protein/209554590?report=genbank&log$=protalign&blast_rank=6&RID=1ZJW93WT01S) |
| 11 | ATCC 33695 | [ZP_03003757.1](http://www.ncbi.nlm.nih.gov/protein/188518220?report=genbank&log$=protalign&blast_rank=3&RID=NRP21EN5014) | [ZP_02556703.2](http://www.ncbi.nlm.nih.gov/protein/188518498?report=genbank&log$=protalign&blast_rank=2&RID=NRMMTAJ2011) | -- | [ZP_03003852.1](http://www.ncbi.nlm.nih.gov/protein/188518339?report=genbank&log$=protalign&blast_rank=2&RID=PC8KDU80011) | [ZP_02557214.2](http://www.ncbi.nlm.nih.gov/protein/188518714?report=genbank&log$=protalign&blast_rank=2&RID=1ZCJH80W01S) | [ZP_03004135.1](http://www.ncbi.nlm.nih.gov/protein/188518713?report=genbank&log$=protalign&blast_rank=2&RID=1ZDT6SU301N) | [ZP_03003818.1](http://www.ncbi.nlm.nih.gov/protein/188518297?report=genbank&log$=protalign&blast_rank=5&RID=1ZGVTRBM012) | [ZP_03003816.1](http://www.ncbi.nlm.nih.gov/protein/188518293?report=genbank&log$=protalign&blast_rank=9&RID=1ZJW93WT01S) |
| 12 | ATCC 33696 | [ZP_03004442.1](http://www.ncbi.nlm.nih.gov/protein/188524422?report=genbank&log$=protalign&blast_rank=1&RID=NRP21EN5014) | [ZP_02557248.2](http://www.ncbi.nlm.nih.gov/protein/188524047?report=genbank&log$=protalign&blast_rank=1&RID=NRN2WWZ5016) | -- | [ZP_03004344.1](http://www.ncbi.nlm.nih.gov/protein/188524306?report=genbank&log$=protalign&blast_rank=2&RID=PC8KDU80011) | [ZP_02557455.2](http://www.ncbi.nlm.nih.gov/protein/188524147?report=genbank&log$=protalign&blast_rank=2&RID=1ZCJH80W01S) | [ZP_03004216.1](http://www.ncbi.nlm.nih.gov/protein/188524146?report=genbank&log$=protalign&blast_rank=2&RID=1ZDT6SU301N) | [ZP_03004375.1](http://www.ncbi.nlm.nih.gov/protein/188524343?report=genbank&log$=protalign&blast_rank=6&RID=1ZGVTRBM012) | [ZP_03004374.1](http://www.ncbi.nlm.nih.gov/protein/188524342?report=genbank&log$=protalign&blast_rank=7&RID=1ZJW93WT01S) |
| 13 | ATCC 33698 | -- | [ZP_02931836.1](http://www.ncbi.nlm.nih.gov/protein/171920572?report=genbank&log$=prottop&blast_rank=6&RID=NRN2WWZ5016) | -- | [ZP_02931886.1](http://www.ncbi.nlm.nih.gov/protein/171920649?report=genbank&log$=protalign&blast_rank=4&RID=PC8KDU80011) | [ZP_02931917.1](http://www.ncbi.nlm.nih.gov/protein/171920695?report=genbank&log$=protalign&blast_rank=2&RID=1ZCJH80W01S) | [ZP_02931894.1](http://www.ncbi.nlm.nih.gov/protein/171920662?report=genbank&log$=protalign&blast_rank=3&RID=1ZDT6SU301N) | [ZP_02932057.1](http://www.ncbi.nlm.nih.gov/protein/171920906?report=genbank&log$=protalign&blast_rank=6&RID=1ZGVTRBM012) | [ZP_02932082.1](http://www.ncbi.nlm.nih.gov/protein/171920938?report=genbank&log$=protalign&blast_rank=6&RID=1ZJW93WT01S) |
| 14 | ATCC 33697 | -- | [ZP_02958269.1](http://www.ncbi.nlm.nih.gov/protein/183508815?report=genbank&log$=protalign&blast_rank=1&RID=NRMMTAJ2011) | -- | [ZP_02957984.1](http://www.ncbi.nlm.nih.gov/protein/183508443?report=genbank&log$=protalign&blast_rank=1&RID=PC8KDU80011) | [ZP_02689822.2](http://www.ncbi.nlm.nih.gov/protein/183508595?report=genbank&log$=protalign&blast_rank=1&RID=1ZCJH80W01S) | [ZP_02958161.1](http://www.ncbi.nlm.nih.gov/protein/183508667?report=genbank&log$=protalign&blast_rank=1&RID=1ZDT6SU301N) | [ZP_02958068.1](http://www.ncbi.nlm.nih.gov/protein/183508547?report=genbank&log$=protalign&blast_rank=3&RID=1ZGVTRBM012) | [ZP_02958127.1](http://www.ncbi.nlm.nih.gov/protein/183508622?report=genbank&log$=protalign&blast_rank=3&RID=1ZJW93WT01S) |

Ser: serovar. *: locus tags and gene annotations for *U. parvum* serovar 3 (ATCC 700970). NCBI reference sequences are given for annotated genes; (+) positive by Southern blot, (-) negative by Southern blot, (ND) not determined, (--) not present in sequenced genomes.

**Fig. S1. Alignment of three putative tyrosine recombinases of *U. parvum* serovar 3 (ATCC 700970 and ATCC 27815^T^).**

The amino acid sequence of RipX shows 37.2 % identity to XerC and 25.7 % identity to CodV, and XerC shows 30 % identity to CodV.

Boxes A, B and C and identical residues > 50 % are based on ([Esposito & Scocca, 1997](http://nar.oxfordjournals.org/content/25/18/3605.full.pdf+html)).

The two conserved, polar residues T and S at positions 19 and 62 resemble the residues T96 and S139 of the described core-binding domain found in λ-Int ([Swalla et al., 2003](http://nar.oxfordjournals.org/content/31/3/805.full.pdf+html))cit_bfcit_af ref_bf(Swalla, 2003 ref_num52)ref_af.

[Esposito D & Scocca JJ (1997) The integrase family of tyrosine recombinases: evolution of a conserved active site domain. Nucleic Acids Res 25: 3605-3614.](http://nar.oxfordjournals.org/content/25/18/3605.full.pdf+html)

[Swalla BM, Gumport RI & Gardner JF (2003) Conservation of structure and function among tyrosine recombinases: homology-based modeling of the lambda integrase core-binding domain. Nucleic Acids Res 31: 805-818.](http://nar.oxfordjournals.org/content/31/3/805.full.pdf+html)


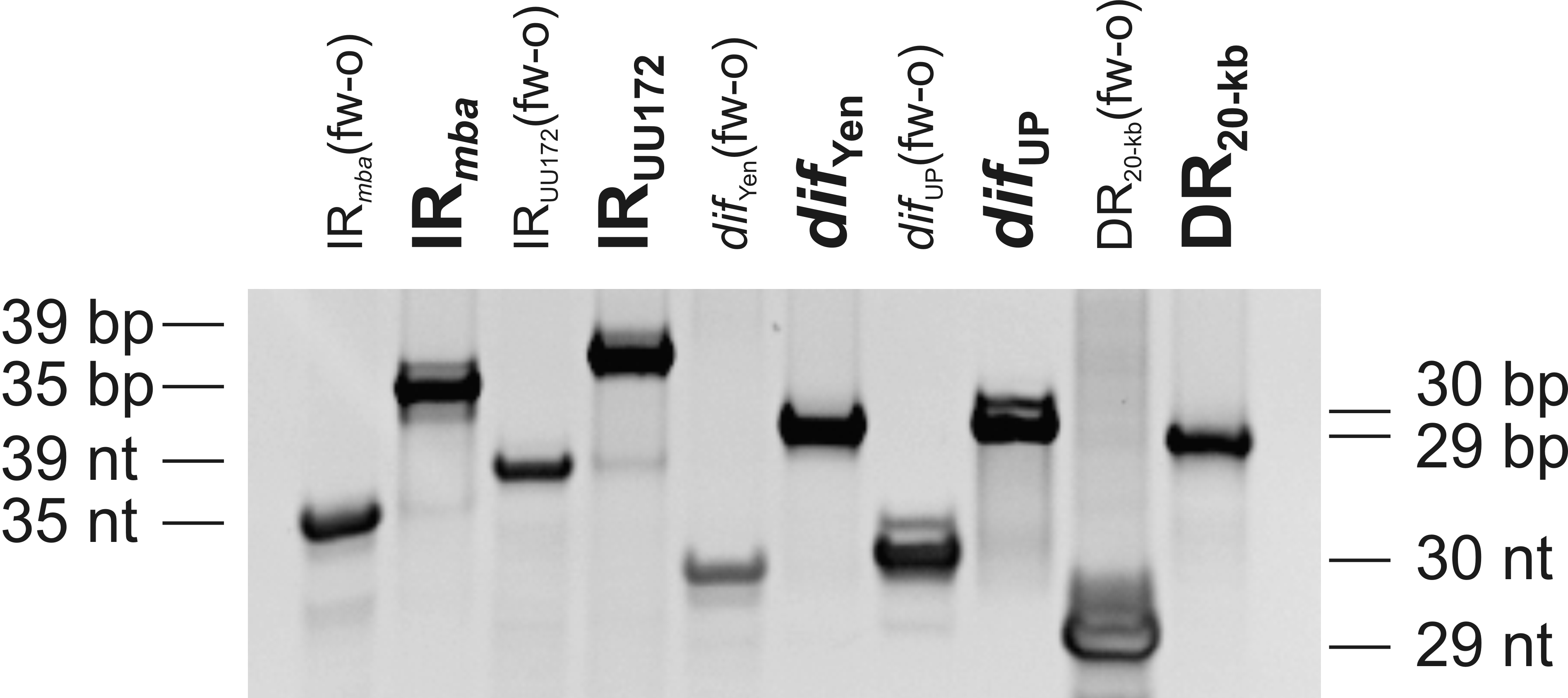


**Fig. S2. Annealed oligonucleotides for EMSA analyses.**

Separation of biotinylated ssDNA forward strand oligonucleotides (fw-o) (100 pmol / lane) and annealed dsDNA products (50 pmol / lane) in 0.5 × TBE / 20% PAGE.


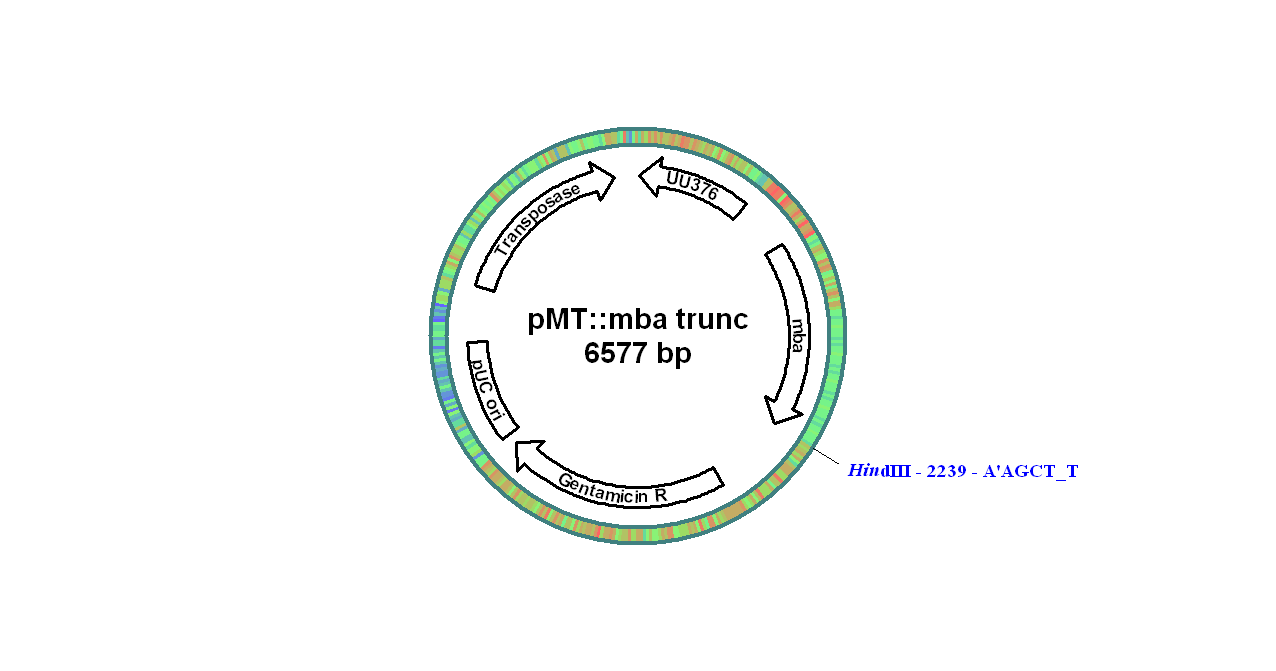


**Fig. S3. Plasmid pMT::*mba*^trunc^**

DNA Sequence:

ACTAGTAAACATTTAACAATTTAGCATTGGTTAATTTAACATCATAATTTGCATTATTTCCATTCATTTTATCTTTTAAATCAATTTCTAAACCATTAAGATTTGATAATTGATTCTTTGTTAATGATTTATGAACTACTTGTAAACTACTATCTTTTTTAATTTCAATATCAAATGTTAATATAGGTTGATTTTCTTCTAAAGAATTTAAAATATCAAAATTTTGTAATTTAACATTAATTTTCTTATTTTCTTTGTTGAATGTCATAAATAATTTTGCATTTTTAACATATGATTTTAACAACTCTTCTTCATTAAATTTGACTTCTTTACCATTTAAAGTTAGTTTTGAAATTTCATAGTCAACATTTGAATTTAAACCACTTAGTTTACCGCTTAATGTTTTAGATGCTTCATCATATTTTAAATCACTTGCTTGAATTGGATTTGTATCAGCTTTCTTAATAACTTCTAAAACAAAATCTTTTTTATTAGCATCTGCTAATTCAAATTTACTAAATGTAACTTCAATAGTAGCAGTATTATTTTTGACTTCACTAATTTTTACACCACTAGCTACAACATCATCTTTTTTAGAATTTTCTGGTTGATCAGCTTGCTGTTCGCTACCACCATTTTCTTTTGGTTCTTCAGTTTTTGGAGCTGGAGTCGGAGATGGTTGTTGGCTTGGAGCTGAAGCTGCTTCAAAGTTCACTTTTTCTGTTTGATAATTCAAATAATAAAACTTATTAAAAATAATATAGATATATAATTAAATTTTTAATCATAATTTATAATAAAAAATATCTAATAATGTTATCGATAATGCAGAAAAATAAAAAAATAAAAAAAATAGTAAAAATTATATAATTAAAAGTGCAAGTGCTAAATAAAAAGTATTTGCAATCTTTATATGTTTTCGTTAAAATTAAAAATTAATTACTGTAGAAATTATGTAAGATTACCAAATCTTAGTGTTCATATTTTTTACATATATTAAATAAAAACAATAAAATGACATATTTTTTATATTAGGAGAATCATAAATGAAATTATTAAAAAATAAAAAATTCTGGGCTATGACATTAGGTGTTACCTTAGTTGGAGCTGGAATAGTTGCTATAGCAGCTTCATGTTCTAATTCAACTGTTAAATCTAAGTTAAGTAACCAATTTGCTAAATCAACAGACGGTAAAAGTTTTTATGCGGTTTACGAAATTGAAAACTTTAAAGATCTAAGTAATGATGATAAAAAATCATTAAGTAACATTGAATTTAATGCTGCACTTACATCAGCTGAAAACAAAACAGAAAGTACACTTGAAAAAGGTCATTTAGTTGGTGAAAAAATTTACGTTAAATTACCTCGTGAACCAAAACCTAATGAACAATTAACTATTATTAGTAAAAGTGGATTAATCAAGACTTCAGGTTTGTTAATATCTGATAATTTGAATTATCAAACAGAAAAAGTGAACTTTGAAACTACACAACCAGGTAAAGAACAACCAGCAGGTAAAGAACAACCAGCAGGTAAAGAACAACCAGCAGGTAAAGAACAACCAGCAGGTAAAGAACAACCAGCAGGTAAAGAACAACCAGCAGGTAAAGAACAACCAGCAGGTAAAGAACAACCAGCAGGTAAAGAACAACCAGCAGGTAAAGAACAACCAGCAGGTAAAGAACAACCAGCAGGTAAAGAACAACCAGCAGGTAAAGAACAACCAGCAGGTAAAGAACAACCAGCAGGTAAAGAACAACCAGCAGGTAAAGAACAACCAGCAGGTAAAGAACAACCAGCAGGTAAAGAACAACCAGCAGGTAAAGAACAACCAGCAGGTAAAGAACAACCAGCAGGTAAAGAACAACCAGCAGGTAAAGAACAACCAGCAGGTAAAGAACAACCAGCAGGTAAAGAACAACCAGCAGGTAAAGAACAACCAGCAGGTAAAGAACAACCAGCAGGTAAAGAACAACCAGCAGGTAAAGAACAACCAGCAGGTAAAGAACAACCAGCAGGTAAAGAACAACCAGCAGGTAAAGAACAACCAGCAGGTAAAGAACAACCAGCAGGTAAAGAACAACCAGCAGGTAAAGAACAACCAGCAGGTAAAGAACAACCAGCAGGTAAAGAACAACCAGCAGGTAAAGAACAACCAGCAGGTAAAGAACAACCAGCAGGTAAAGAACAACCAGCAGGTAAAGAACAACCAGCAGGTAAAGAAACTACTGGAAAATAAGCTTTTTAGGATGAATGGATTTATTCTTCAAGAAAATACATCAATTTTGATAAGTAGAAATGGTAAAAACATTGTATAGCATTTTACACAGGAGTCTGGACTTGACTGAGTTTATGGAAGAAGTTTTAATTGATGATAATATGGTTTTTGATATTGATAATTTAAAAGGATTTCTTAATGATACCAGTTCATTTGGGTTTATAGCTAAAGAAAATAATAAAATTATAGGATTTGCATATTGCTATACACTTTTAAGACCTGATGGAAAAACAATGTTTTATTTACACTCAATAGGAATGTTACCTAACTATCAAGACAAAGGTTATGGTTCAAAATTATTATCTTTTATTAAGGAATATTCTAAAGAGATTGGTTGTTCTGAAATGTTTTTAATAACTGATAAAGGTAATCCTAGAGCTTGCCATGTATATGAAAAATTAGGTGGTAAAAATGATTATAAAGATGAAATAGTATATGTATATGATTATGAAAAAGGTGATAAATAAATGAATATAGTTGAAAATGAAATATGTATAAGAACTTTAATAGATGATGATTTTCCTTTGATGTTAAAATGGTTAACTGATGAAAGAGTATTAGAATTTTATGGTGGTAGAGATAAAAAATATACATTAGAATCATTAAAAAAACATTATACAAAGCCTTGGGAAGATGAAGTTTTTAGAGTAATTATTGAATATAACAATGTTCCTATTGGATATGGACAAATATATAAAATGTATGATGAGTTATATACTGATTATCATTATCCAAAAACTGATGAGATAGTCTATGGTATGGATCAATTTATAGGAGAGCCAAATTATTGGAGTAAAGGAATTGGTACAAGATATATTAAATTGATTTTTGAATTTTTGAAAAAAGAAAGAAATGCTAATGCAGTTATTTTAGACCCTCATAAAAATAATCCAAGAGCAATAAGGGCATACCAAAAATCTGGTTTTAGAATTATTGAAGATTTGCCAGAACATGAATTACACGAGGGCAAAAAAGAAGATTGTTATTTAATGGAATATAGATATGATGATAATGCCACAAATGTTAAGGCAATGAAATATTTAATTGAGCATTACTTTGATAATTTCAAAGTAGATAGTATTGAAATAATCGGTAGTGGTTATGATAGTGTGGCATATTTAGTTAATAATGAATACATTTTTAAAACAAAATTTAGTACTAATAAGAAAAAAGGTTATGCAAAAGAAAAAGCAATATATAATTTTTTAAATACAAATTTAGAAACTAATGTAAAAATTCCTAATATTGAATATTCGTATATTAGTGATGAATTATCTATACTAGGTTATAAAGAAATTAAAGGAACTTTTTTAACACCAGAAATTTATTCTACTATGTCAGAAGAAGAACAAAATTTGTTAAAACGAGATATTGCCAGTTTTTTAAGACAAATGCACGGTTTAGATTATACAGATATTAGTGAATGTACTATTGATAATAAACAAAATGTATTAGAAGAGTATATATTGTTGCGTGAAACTATTTATAATGATTTAACTGATATAGAAAAAGATTATATAGAAAGTTTTATGGAAAGACTAAATGCAACAACAGTTTTTGAGGGTAAAAAGTGTTTATGCCATAATGATTTTAGTTGTAATCATCTATTGTTAGATGGCAATAATAGATTAACTGGAATAATTGATTTTGGAGATTCTGGAATTATAGATGAATATTGTGATTTTATATACTTACTTGAAGATAGTGAAGAAGAAATAGGAACAAATTTTGGAGAAGATATATTAAGAATGTATGGAAATATAGATATTGAGAAAGCAAAAGAATATCAAGATATAGTTGAAGAATATTATCCTATTGAAACTATTGTTTATGGAATTAAAAATATTAAACAGGAATTTATCGAAAATGGTAGAAAAGAAATTTATAAAAGGACTTATAAAGATTAATAAAGATCTACGAAGGCATGACCAAAATCCCTTAACGTGAGTTTTCGTTCCACTGAGCGTCAGACCCCGTAGAAAAGATCAAAGGATCTTCTTGAGATCCTTTTTTTCTGCGCGTAATCTGCTGCTTGCAAACAAAAAAACCACCGCTACCAGCGGTGGTTTGTTTGCCGGATCAAGAGCTACCAACTCTTTTTCCGAAGGTAACTGGCTTCAGCAGAGCGCAGATACCAAATACTGTTCTTCTAGTGTAGCCGTAGTTAGGCCACCACTTCAAGAACTCTGTAGCACCGCCTACATACCTCGCTCTGCTAATCCTGTTACCAGTGGCTGCTGCCAGTGGCGATAAGTCGTGTCTTACCGGGTTGGACTCAAGACGATAGTTACCGGATAAGGCGCAGCGGTCGGGCTGAACGGGGGGTTCGTGCACACAGCCCAGCTTGGAGCGAACGACCTACACCGAACTGAGATACCTACAGCGTGAGCTATGAGAAAGCGCCACGCTTCCCGAAGGGAGAAAGGCGGACAGGTATCCGGTAAGCGGCAGGGTCGGAACAGGAGAGCGCACGAGGGAGCTTCCAGGGGGAAACGCCTGGTATCTTTATAGTCCTGTCGGGTTTCGCCACCTCTGACTTGAGCGTCGATTTTTGTGATGCTCGTCAGGGGGGCGGAGCCTATGGAAAAACGCCAGCAACGCGGCCTTTTTACGGTTCCTGGCCTTTTGCTGGCCTTTTGCTCACATGTTCTTTCCTGCGTTATCCCCTGATTCTGTGGATAACCGTATTACCGCCTTTGAGTGAGCTGATACCGCTCGCCGCAGCCGAACGACCGAGCGCAGCGAGTCAGTGAGCGAGGAAGCGGAAGAGCGCCCAATACGCAAACCGCCTCTCCCCGCGCGTTGGCCGATTCATTAATGCACGCTAGCATTTAAATGGTACCCTTTTACACAATTATACGGACTTTATCCTGCAGGGGCCCAATTGTGTAAAAGTAAAAAGGCCATATAACAGTCCTTTTACGGTACAATGTTTTTAACGACAAAAACATACCCAGGAGGACTTTTACATGACCCAAGTACATTTTACACTGAAAAGCGAAGAGATTCAAAGCATTATTGAATATTCTGTAAAGGATGACGTTTCTAAAAATATTTTAACAACGGTATTTAATCAACTAATGGAAAATCAACGAACAGAATATATTCAAGCAAAAGAATATGAACGAACAGAAAACCGACAAAGTCAACGAAATGGCTATTATGAGCGCAGCTTTACGACACGTGTAGGCACGCTAGAATTAAAAGTACCCAGAACACGTGATGGCCATTTTTCACCCACAGTGTTTGAACGTTATCAACGAAACGAAAAAGCCCTCATGGCTTCAATGTTGGAAATGTATGTATCAGGCGTTTCAACTCGTAAAGTATCAAAAATTGTGGAAGAACTTTGTGGTAAATCCGTCTCTAAGTCCTTCGTTTCTAGCTTAACAGAACAGCTAGAACCTATGGTTAACGAGTGACAGAATCGTTTATTATCAGAAAAAAATTATCCTTACTTAATGACCGATGTACTCTATATAAAAGTACGAGAAGAAAATCGAGTACTCTCAAAAAGCTGTCATATAGCGATTGGAATAACCAAAGATGGCGACCGTGAAATTATCGGCTTCATGATTCAAAGTGGCGAAAGCGAAGAGACCTGGACAACATTTTTTGAATACCTAAAAGAACGCGGTTTACAAGGTACGGAACTCGTTATTTCTGATGCGCACAAAGGATTAGTCTCTGCCATTAGAAAATCCTTCACCAACGTAAGTTGGCAAAGATGCCAAGTTCACTTCCTAAGAAATATCTTTACCACCATTCCTAAAAAAAATTCAAAATCTTTCAGAGAAGCTGTTAAAGGAATTTTTAAGTTCACAGATATTAACTTAGCGCGTGAGGCTAAAAATCGATTGATTCATGATTATATCGATCAACCAAAATATTCAAAGGCTTGCGCATCATTGGATGATGGATTCGAAGACGCCTTTCAATATACCGTACAAGGAAATTCCCACAATCGACTAAAGAGTACCAATCTAATTGAACGACTGAATCAAGAAGTACGCAGAAGAGAAAAGATTATTCGCATCTTCCCCAATCAAACATCAGCCAATCGCTTAATTGGAGCCGTTCTTATGGACCTACATGATGAATGGATTTATTCTTCAAGAAAATACATCAATTTTGATAAGTAGAAATGGTAAAAACATTGTATAGCATTTTACACAGGAGTCTGGACTTGACTCACTTCCTTTATTATTTTTCATTTTTTTGACCTCGAGGGGGGGCCCACCATACAGCTGACGATAAAGTCCGTATAATTGTGTAAAAACCCATAGCTTTGGACACAC

The vector map was drawn with software pDRAW32; ACACLONE Software: [^http://www.acaclone.com^](http://www.acaclone.com/).


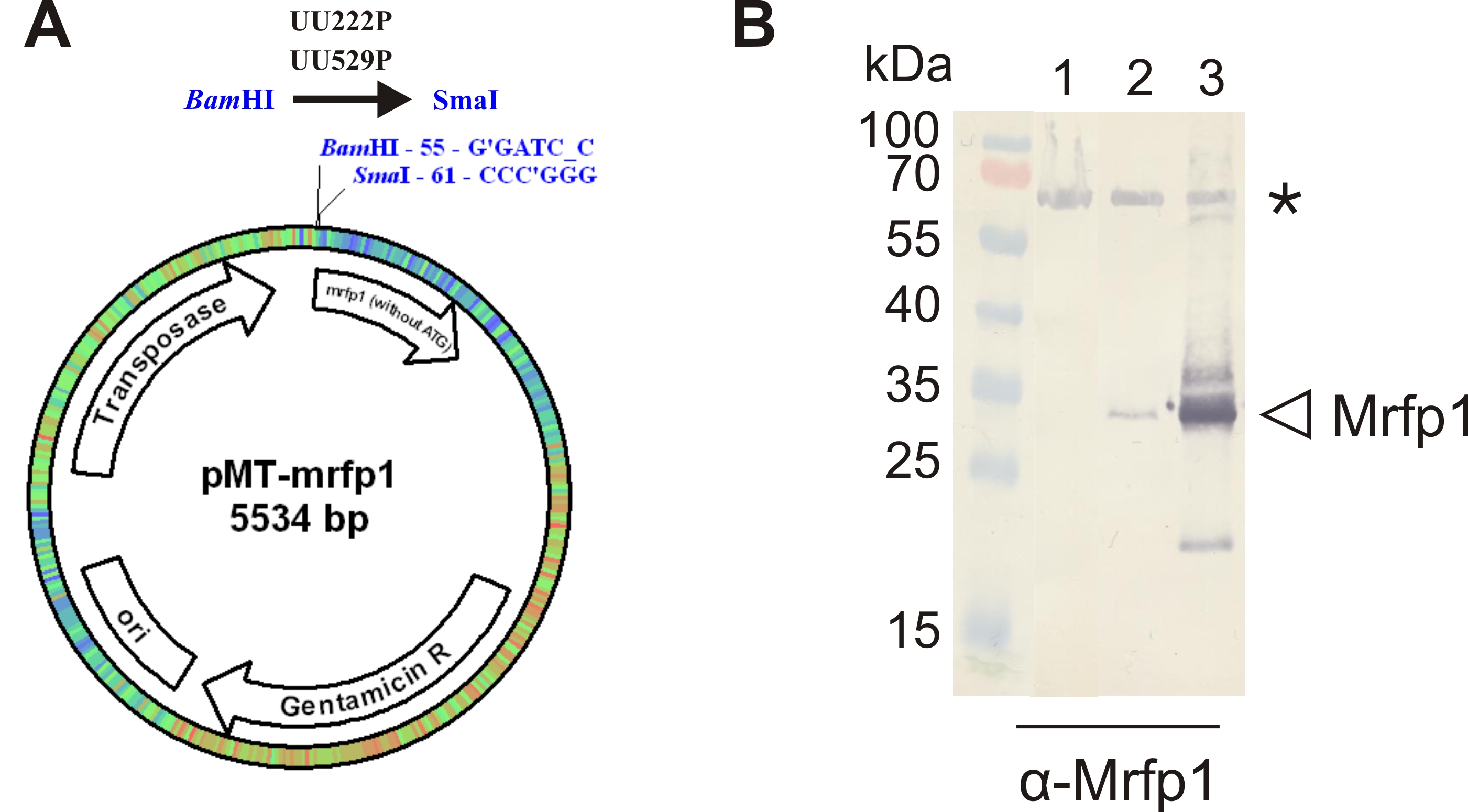
**Fig. S4. Promoter fusion and Mrfp1 expression from *Ureaplasma* promoters in *Mycoplasma pneumoniae* M129.**

Upstream regions UU222P and UU529P of recombinase encoding genes UU222 and UU529 were PCR amplified from genomic DNA of *U. parvum* serovar 3 (details see below) and ligated into the *Bam*HI and *Sma*I restriction sites of plasmid pMT-*mrfp1* ([Zimmerman and Herrmann, 2005](http://www.ncbi.nlm.nih.gov/pubmed/16260096)) (**A**). The *Sma*I sequence (CCCGGG) was used as linker between a short 5’ sequence of either recombinase gene and the second codon of the *mrfp1* gene ([Campbell et al., 2002](http://www.pnas.org/content/99/12/7877.full.pdf+html)), yielding plasmids pMT-UU222P::*mrfp1* and pMT-UU529P::*mrfp1*. *M. pneumoniae* M129 was transformed by electroporation with the plasmids and subcloned.

(**B**) Western blot analysis was carried out with total protein of *M. pneumoniae* M129 (MP) (lane 1), an MP clone transformed with pMT-UU222P::*mrfp1* (lane 2) or with pMT-UU529P::*mrfp1* (lane 3) using polyclonal antibodies against the monomeric red fluorescent protein Mrfp1 ([Zimmerman and Herrmann, 2005](http://www.ncbi.nlm.nih.gov/pubmed/16260096)). The asterisk indicates a background band. Mrfp1 expression was clearly stronger when fused to UU529P, indicating strong promoter activity from this region.

Details for PCR amplified, putative promoter regions:

## Product: UU222P

## Comprises 295 bp upstream of UU222 and the first five codons of UU222; the ATG start codon is indicated in bold and italic of the reverse primer.

Forward primer: 5’-TTA**GGATCC**TTCTGATGATGCAAAATTAT-3’

## Reverse primer: 5’-TG**CCCGGG**AATAAAATCTTT***CAT***ATTATC-3’

## Product: UU529P:

## Comprises 275 bp upstream of UU529 and the first seven codons of UU529; the ATG start codon is indicated in bold and italic of the reverse primer.

Forward primer: 5’-AT**GGATCC**GATGTTGTGCTCCAATTCCAG-3’

Reverse primer: 5’-TG**CCCGGG**ATAATTTATAAATTTTTT***CAT***-3’

[Campbell, RE, Tour, O, Palmer, AE, Steinbach, PA, Baird, GS, Zacharias, DA & Tsien, RY (2002) A monomeric red fluorescent protein. Proc. Natl. Acad. Sci. USA 99: 7877–7882.](http://www.pnas.org/content/99/12/7877.full.pdf+html)

[Zimmerman CU & Herrmann, R (2005) Synthesis of a small, cysteine-rich, 29 amino acids long peptide in Mycoplasma pneumoniae. FEMS Microbiol Lett 253: 315-321.](http://www.ncbi.nlm.nih.gov/pubmed/16260096)

The vector map was drawn with software pDRAW32; ACACLONE Software: [^http://www.acaclone.com^](http://www.acaclone.com/).

**Fig. S5. Growth curve of MBP::CodV expressing *E. coli*.**

*E. coli* DH10B containing expression vector pMAL-c2X::UU529 were grown in 100 ml broth (20 g L^-1^ trypton, 10 g L^-1^ yeast extract, 5 g L^-1^ NaCl) at 37°C. Cells were split into two Erlenmeyer Flasks (each 50 ml) when the optical density (OD_600_) had reached 0.45 and protein expression was induced by adding IPTG at a final concentration of 0.5 mM. Cell titers were determined by plating dilutions on growth agar before induction and every hour post induction (**Table S2**). The cell titer dropped from 9 × 10^7^ cfu / ml to 3 × 10^6^ cfu / ml after 4 hours induction with IPTG, indicating CodV toxicity to *E. coli*.

### Table S2. Growth of MBP::CodV expressing *E. coli*.

| **Tine** | **0h** | **1h** | **2h** | **3h** | **4h** |
| --- | --- | --- | --- | --- | --- |
| cfu/ml (- IPTG) | 8.7 × 10^7^ | 6.2 × 10^8^ | 1.2 × 10^9^ | 2.5 × 10^9^ | 2.0 × 10^9^ |
| cfu/ml (+ IPTG) | 8.7 × 10^7^ | 2.4 × 10^6^ | 1.0 × 10^7^ | 4.2 × 10^6^ | 3.2 × 10^6^ |
| OD_600_ (- IPTG) | 0.45 | 1.19 | 2.52 | 3.65 | 4.1 |
| OD_600_ (+ IPTG) | 0.45 | 0.56 | 0.55 | 0.53 | 0.5 |


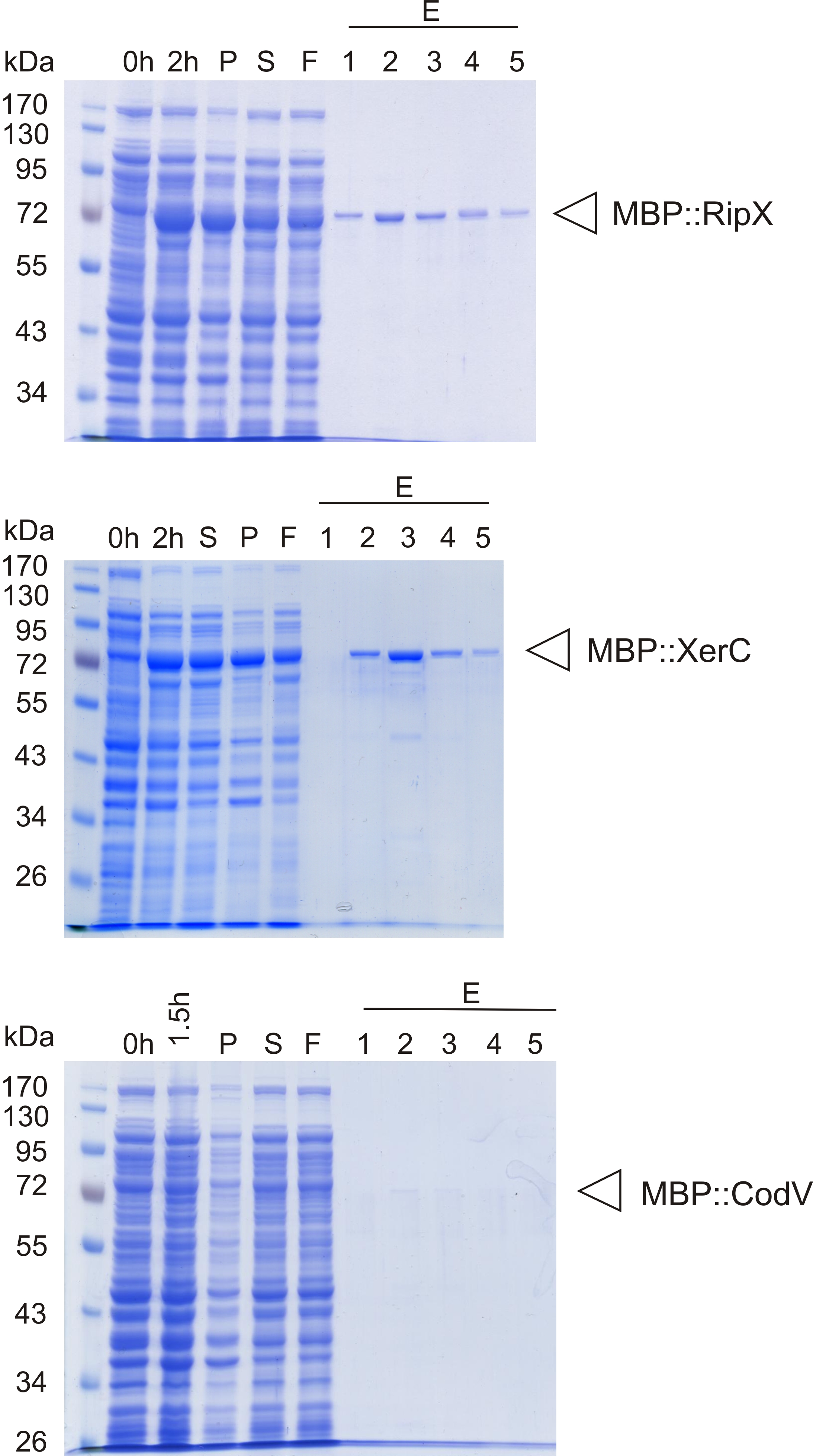


**Fig. S6. Protein expression and purification of MBP fusion proteins.**

Expression and purification by affinity chromatography of maltose binding protein (MBP) fusions of RipX, XerC and CodV from *U. parvum* serovar 3. Proteins were separated in a 10 % SDA/PAGE gel and stained with Coomassie Blue. Labeling: 0h, total cell protein before IPTG induction; 2h, total cell protein after IPTG induction for 2h; S, protein from soluble (supernatant) lysate fraction; P, insoluble (pellet) lysate fraction after centrifugation; F, flow through fraction; E 1-5, elution fractions.


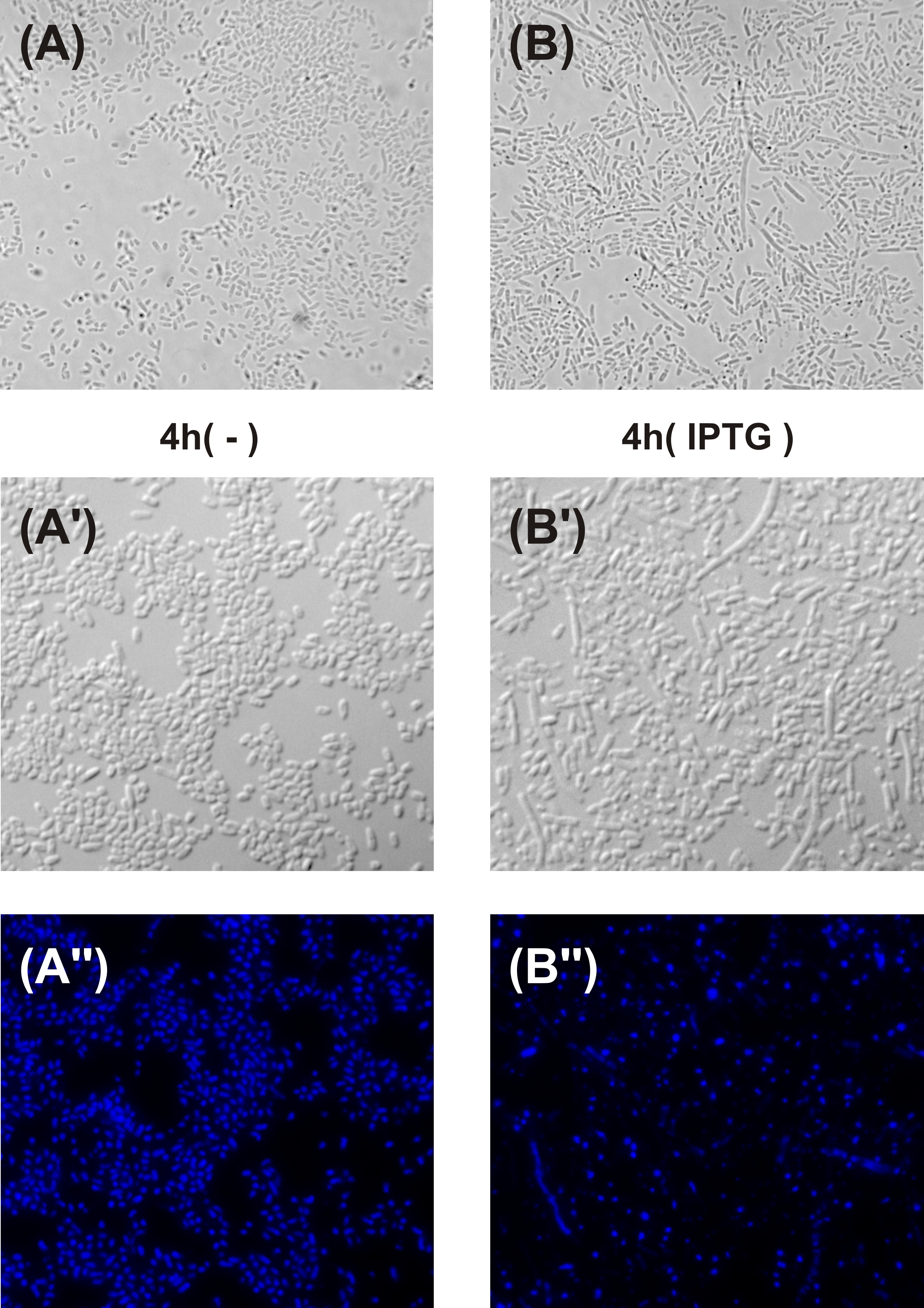
**Fig. S7. Cell morphology of MBP::CodV expressing *E. coli*.**

*E. coli* DH10B containing expression vector pMAL-c2X::UU529 were grown in broth (20 g L^-1^ trypton, 10 g L^-1^ yeast extract, 5 g L^-1^ NaCl) at 37°C. Protein expression of fusion protein MBP::CodV was either uninduced (**A**) or induced (**B**) for four hours with 0.5 mM IPTG before cells were microscopically observed. **A** and **B**, 600 × magnification;

**A’**, **A’’**, **B’** and **B’’**, 10,000 × magnification; **A’’** and **B’’**, DAPI stained DNA. Filamentous growth and loss of DNA content in MBP::CodV expressing cells indicates CodV toxicity for *E. coli*.


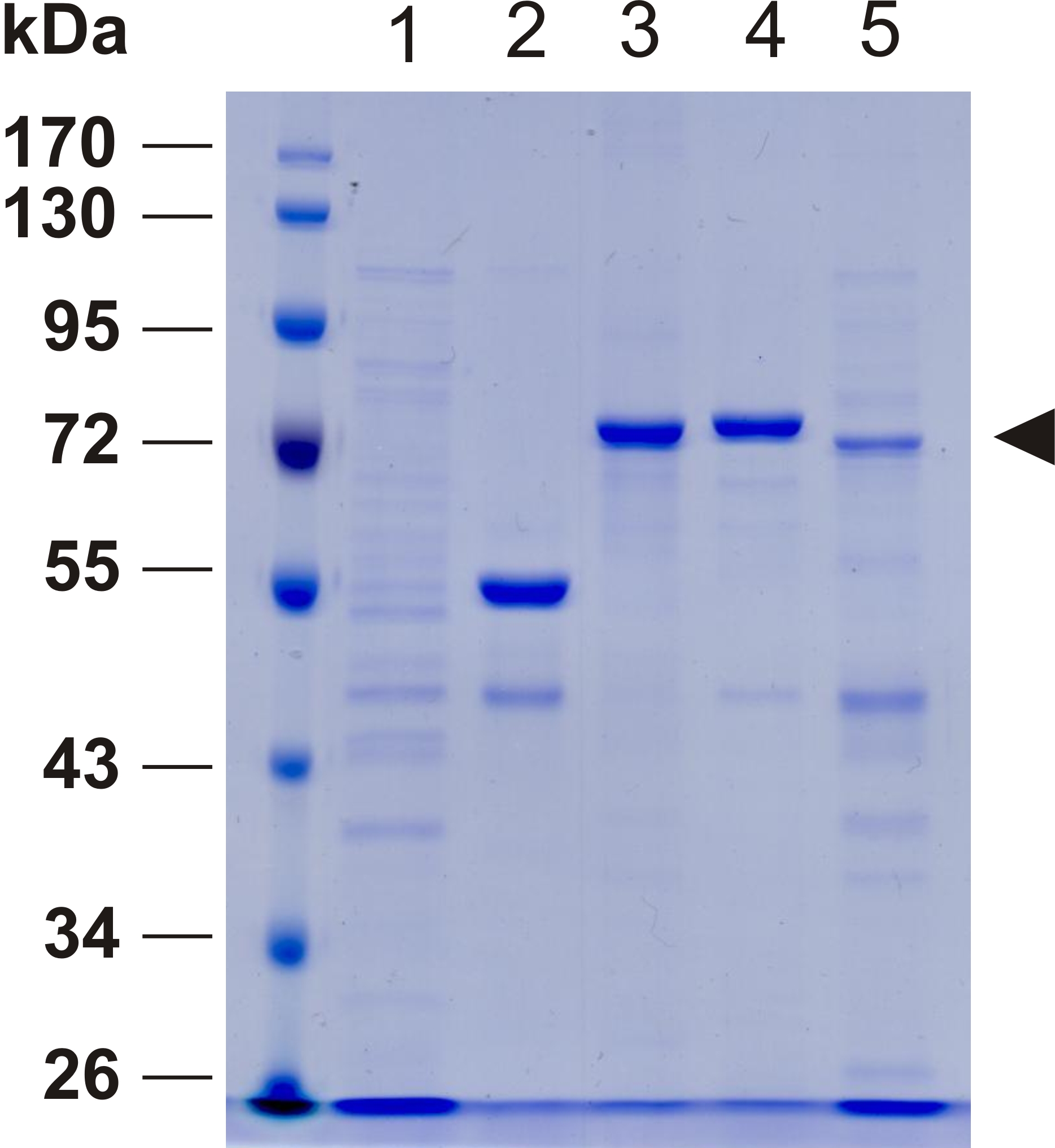


**Fig. S8. Protein preparations for EMSA analyses.**

Separation of protein preparations (1 µg / lane) in a 10 % SDS/PAGE gel. Lanes: **1**, soluble fraction of *E. coli* DH10B, **2**, MBP, **3**, MBP::RipX, **4**, MBP::XerC and **5**, MBP::CodV (1.6 µg). Proteins were stained with Coomassie Blue. ◄: indicates the location of MBP fusion protein.

**Table S3. Features of proteins used for EMSA analyses.**

| Protein Name | length (amino acids) | molecular weight (kilodaltons) |
| --- | --- | --- |
| MBP | 462 | 50.84 |
| MBP::RipX | 648 | 73.85 |
| MBP::XerC | 651 | 74.24 |
| MBP::CodV | 644 | 72.78 |


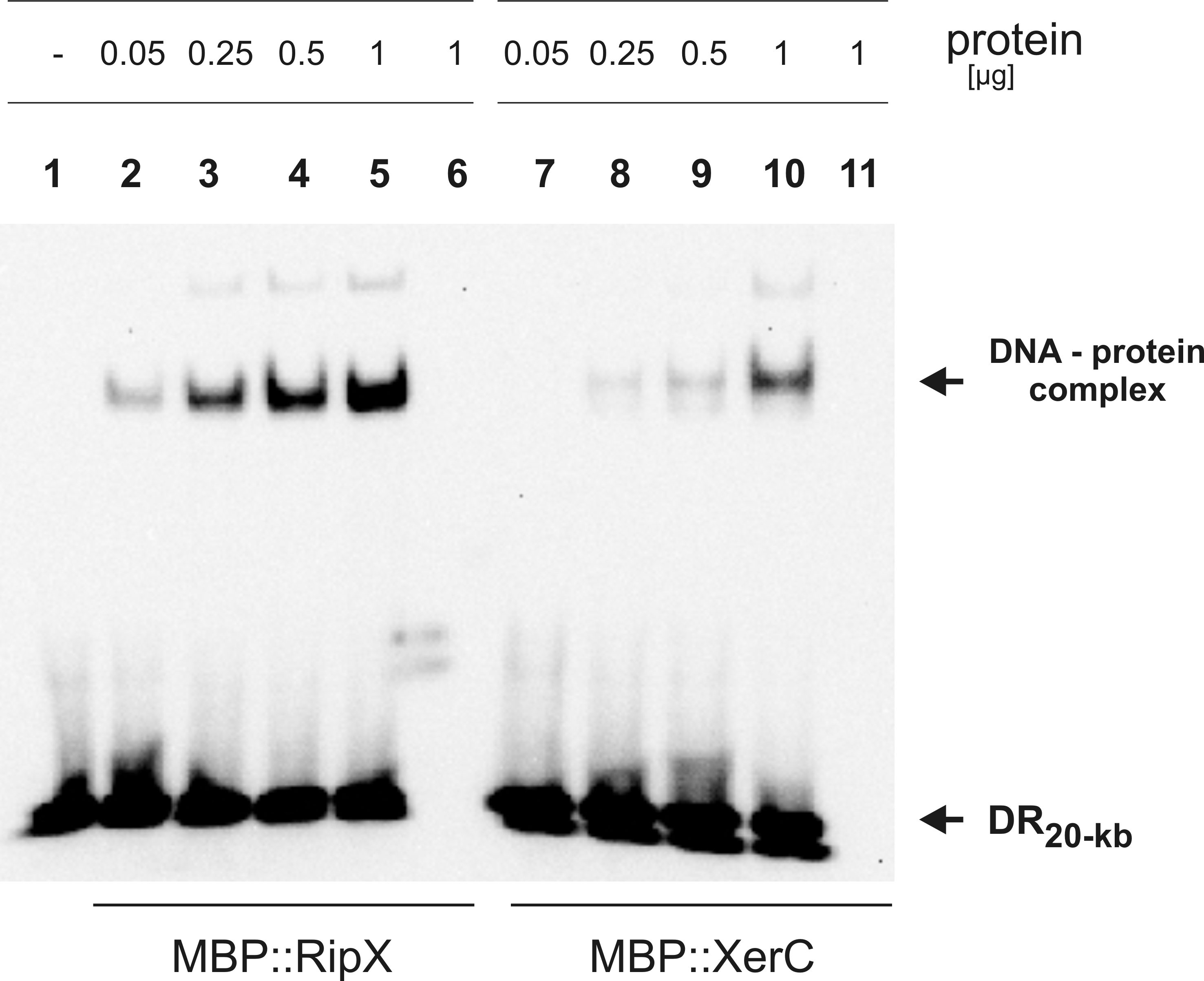


**Fig. S9. Protein concentration-dependent binding of MBP::RipX and MBP::XerC to DR_20-kb_.**

EMSA analysis using purified MBP::RipX and MBP::XerC fusions at different concentrations and biotin labeled substrate DR_20-kb_.

Lane 1, DR_20-kb_

Lane 2, DR_20-kb_ + MBP::RipX (50 ng)

Lane 3, DR_20-kb_ + MBP::RipX (250 ng)

Lane 4, DR_20-kb_ + MBP::RipX (500 ng)

Lane 5, DR_20-kb_ + MBP::RipX (1 µg)

Lane 6, MBP::RipX (1µg)

Lane 7, DR_20-kb_ + MBP::XerC (50 ng)

Lane 8, DR_20-kb_ + MBP::XerC (250 ng)

Lane 9, DR_20-kb_ + MBP::XerC (500 ng)

Lane 10, DR_20-kb_ + MBP::XerC (1 µg)

Lane 11, MBP::XerC (1 µg)


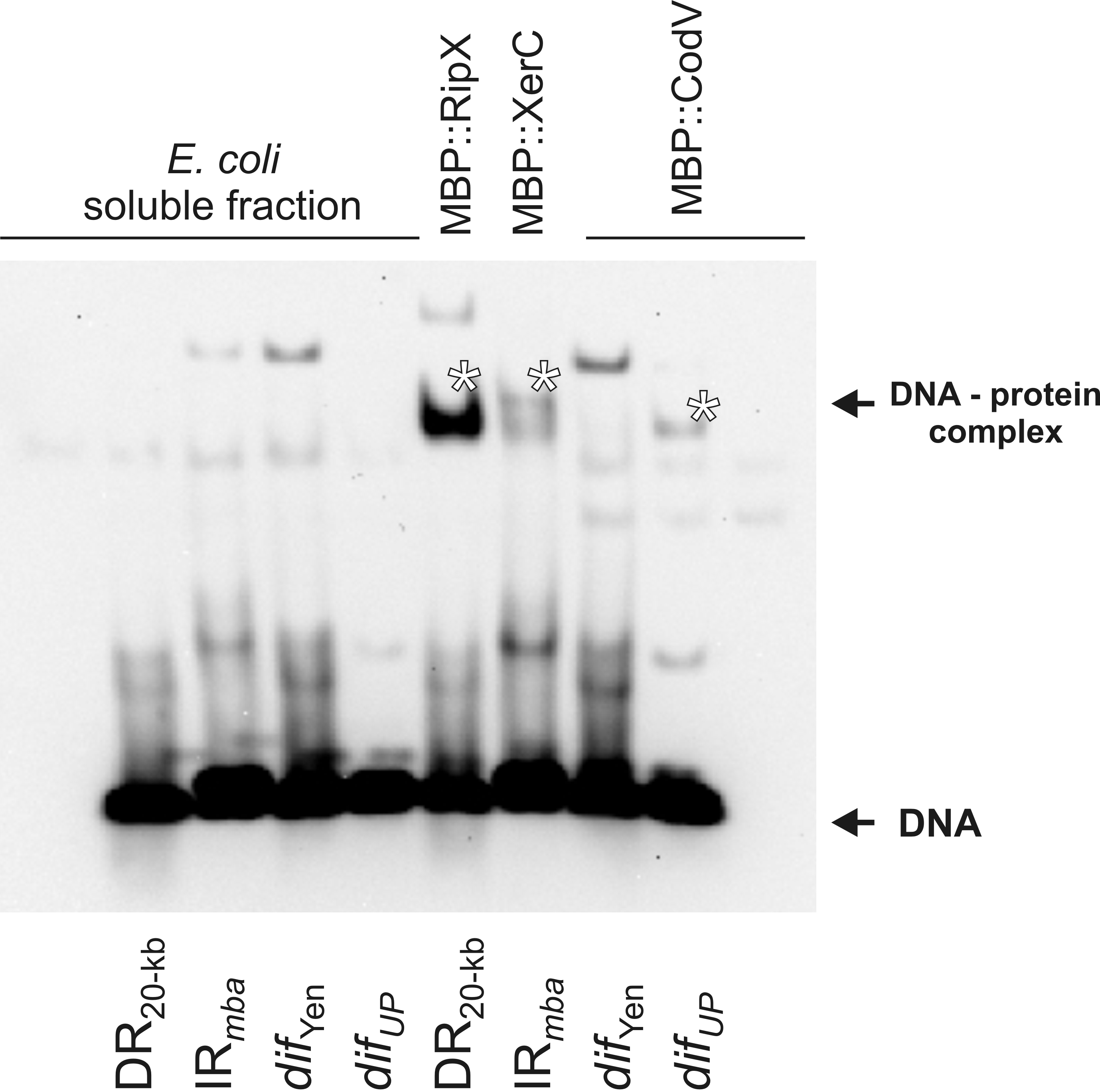


**Fig. S10. Protein-DNA interaction.**

Protein-DNA interaction of purified proteins and the soluble protein fraction of *E. coli* DH10B with different biotinylated substrate DNAs. For each reaction, 250 ng protein was used (except for MBA::CodV, where 400 ng was). Specific interactions of recombinant fusion-proteins with substrate DNA are labeled with an asterisk. Note that the soluble protein fraction of the *E. coli* lysate is not interacting with DR_20-kb_ and *dif_UP_*, however, interacts with IR*_mba_* and *dif*_Yen_; these background bands run above the specific interactions of recombinant fusion-proteins with substrate DNA (labeled with an asterisk). Background bands deriving from protein preparations are visible in the first lane (*E. coli* soluble protein fraction) and the last lane (MBP::CodV).


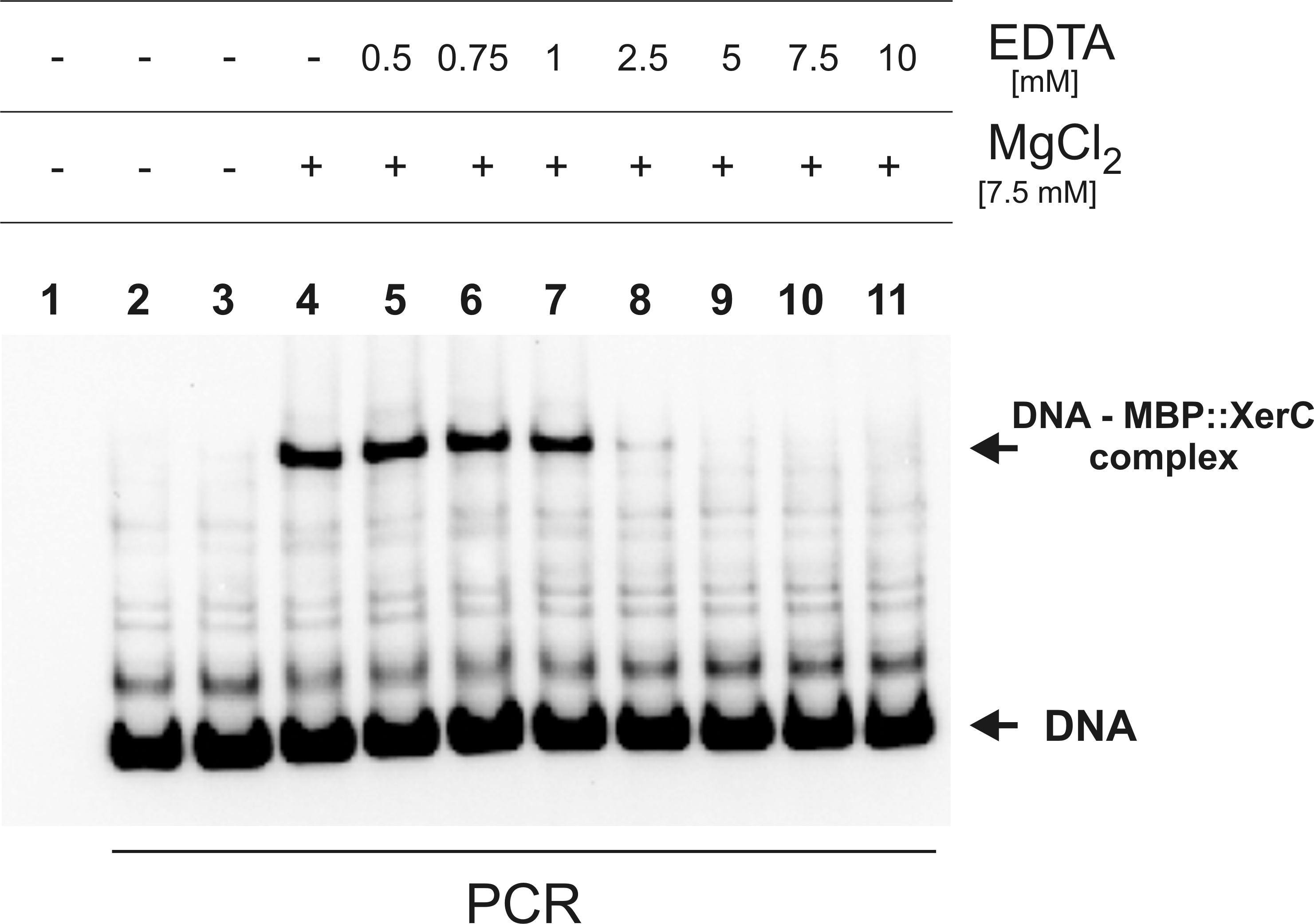


**Fig. S11. Inhibition of XerC binding to IR*_mba_* by EDTA.**

Magnesium-dependent binding of MBP::XerC to substrate DNA *in vitro*. EMSA analysis using a purified MBP fusion of XerC (250 ng / reaction) and a biotinylated PCR product of 145 bp containing one inverted repeat IR*_mba_*.

Lane 1, MBP::XerC

Lane 2, PCR

Lane 3, PCR + MBP::XerC

Lane 4, PCR + MBP::XerC + MgCl_2_ (7.5 mM)

Lane 5, PCR + MBP::XerC + MgCl_2_ (7.5 mM) + EDTA (0.5 mM)

Lane 6, PCR + MBP::XerC + MgCl_2_ (7.5 mM) + EDTA (0.75 mM)

Lane 7, PCR + MBP::XerC + MgCl_2_ (7.5 mM) + EDTA (1 mM)

Lane 8, PCR + MBP::XerC + MgCl_2_ (7.5 mM) + EDTA (2.5 mM)

Lane 9, PCR + MBP::XerC + MgCl_2_ (7.5 mM) + EDTA (5 mM)

Lane 10, PCR + MBP::XerC + MgCl_2_ (7.5 mM) + EDTA (7.5 mM)

Lane 11, PCR + MBP::XerC + MgCl_2_ (7.5 mM) + EDTA (10 mM)


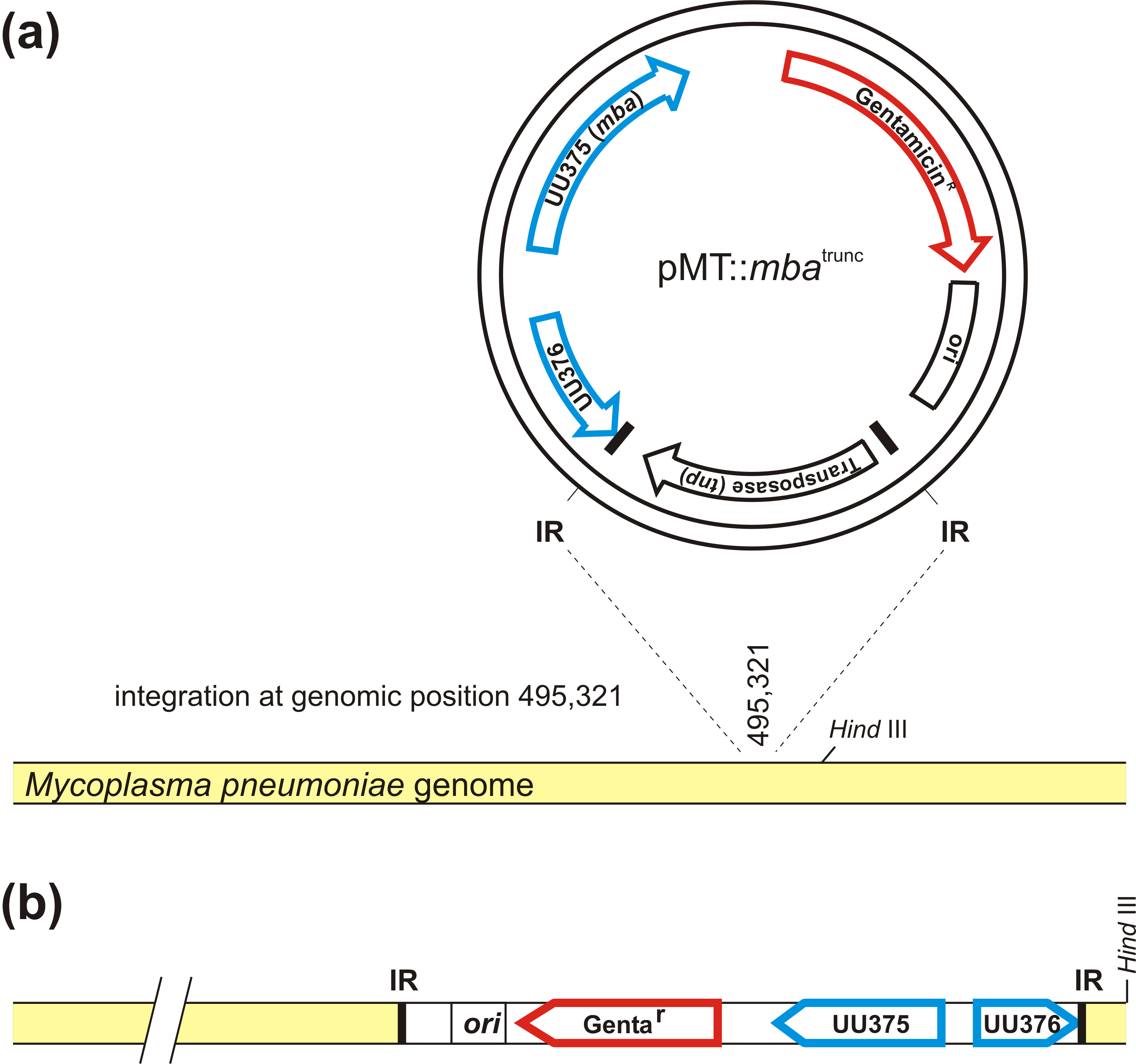


### Fig. S12. Integration of *mba*^trunc^ into the *Mycoplasma pneumoniae* chromosome.

Schematic illustration of partial integration of the modified transposon vector pMT::*mba*^trunc^ into the chromosome of *Mycoplasma pneumoniae*. Integration occurred at the inverted repeats (IR), which flank the transposase gene *tnp*. (**a**) Illustrates the plasmid pMT::*mba*^trunc^ and the chromosomal position of integration (dotted line). (**b**) Illustrates the integration of *mba*^trunc^ in the chromosome via the IR sequences. The transposase gene *tnp* was lost during the integration process.


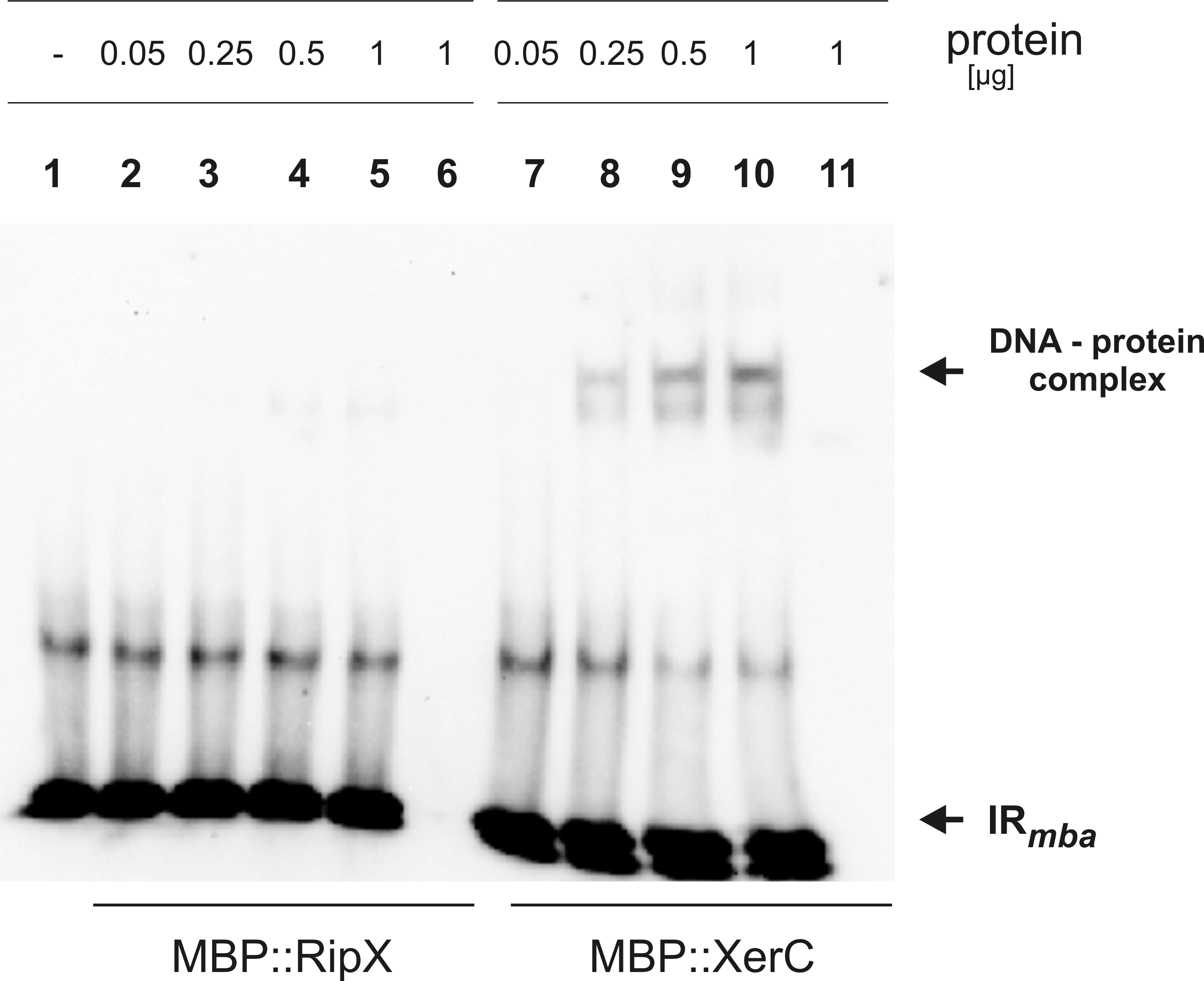


**Fig. S13. Protein concentration-dependent binding of MBP::RipX and MBP::XerC to IR*_mba_*.**

EMSA analysis using purified MBP::RipX and MBP::XerC fusions at different concentrations and biotin labeled substrate IR*_mba_*.

Lane 1, IR*_mba_*

Lane 2, IR*_mba_* + MBP::RipX (50 ng)

Lane 3, IR*_mba_* + MBP::RipX (250 ng)

Lane 4, IR*_mba_* + MBP::RipX (500 ng)

Lane 5, IR*_mba_* + MBP::RipX (1 µg)

Lane 6, MBP::RipX (1µg)

Lane 7, IR*_mba_* + MBP::XerC (50 ng)

Lane 8, IR*_mba_* + MBP::XerC (250 ng)

Lane 9, IR*_mba_* + MBP::XerC (500 ng)

Lane 10, IR*_mba_* + MBP::XerC (1 µg)

Lane 11, MBP::XerC (1 µg)


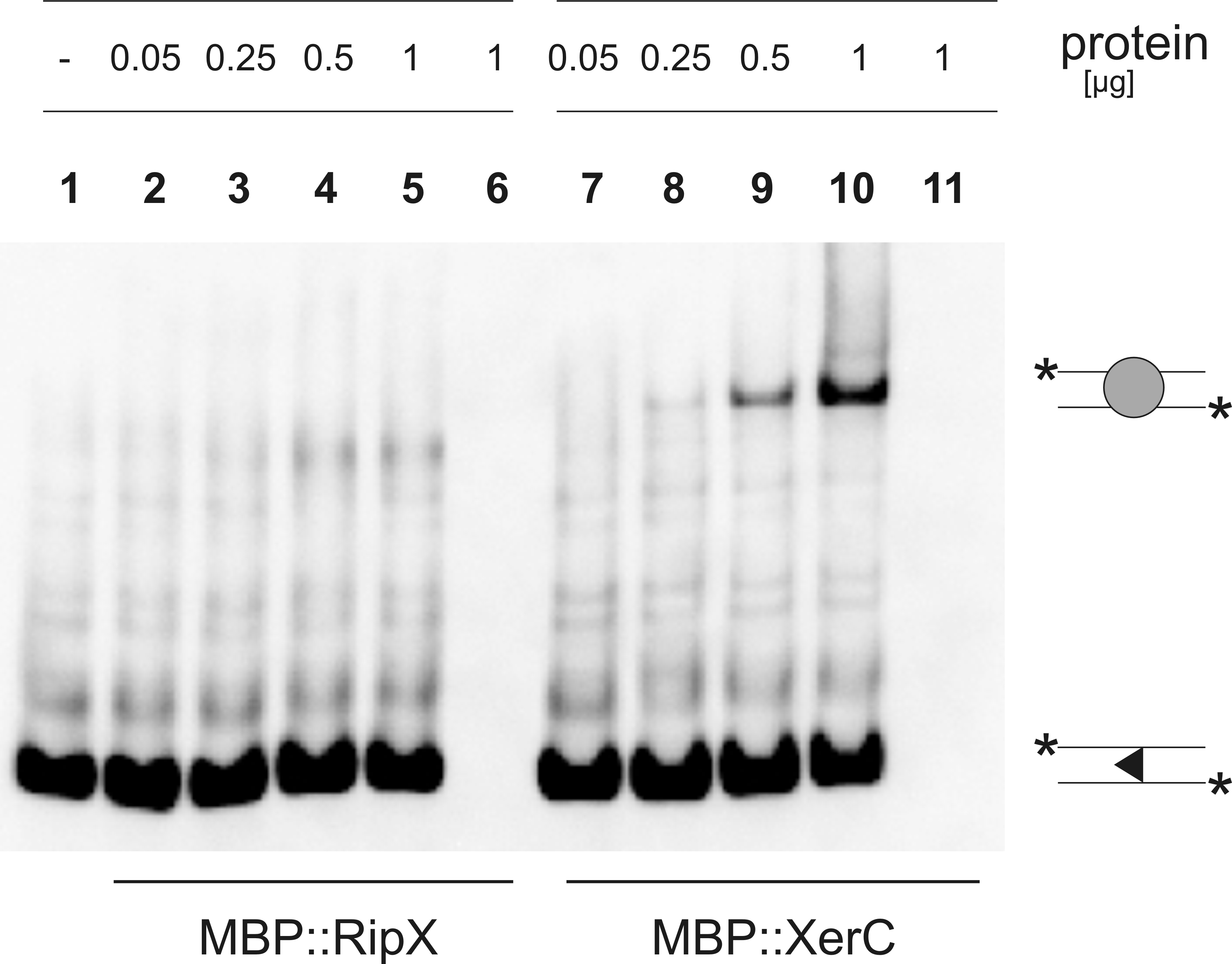


**Fig. S14. Protein concentration-dependent binding of MBP::XerC to IR*_mba_* on a 145-bp PCR product.**

EMSA analysis using purified MBP::RipX and MBP::XerC fusion (●) and a biotinylated (*) PCR product of 145 bp containing one inverted repeat (◄) (IR*_mba_*)

Lane 1, PCR

Lane 2, PCR + MBP::RipX (50 ng)

Lane 3, PCR + MBP::RipX (250 ng)

Lane 4, PCR + MBP::RipX (500 ng)

Lane 5, PCR + MBP::RipX (1 µg)

Lane 6, MBP::RipX (1µg)

Lane 7, PCR + MBP::XerC (50 ng)

Lane 8, PCR + MBP::XerC (250 ng)

Lane 9, PCR + MBP::XerC (500 ng)

Lane 10, PCR + MBP::XerC (1 µg)

Lane 11, MBP::XerC (1 µg)


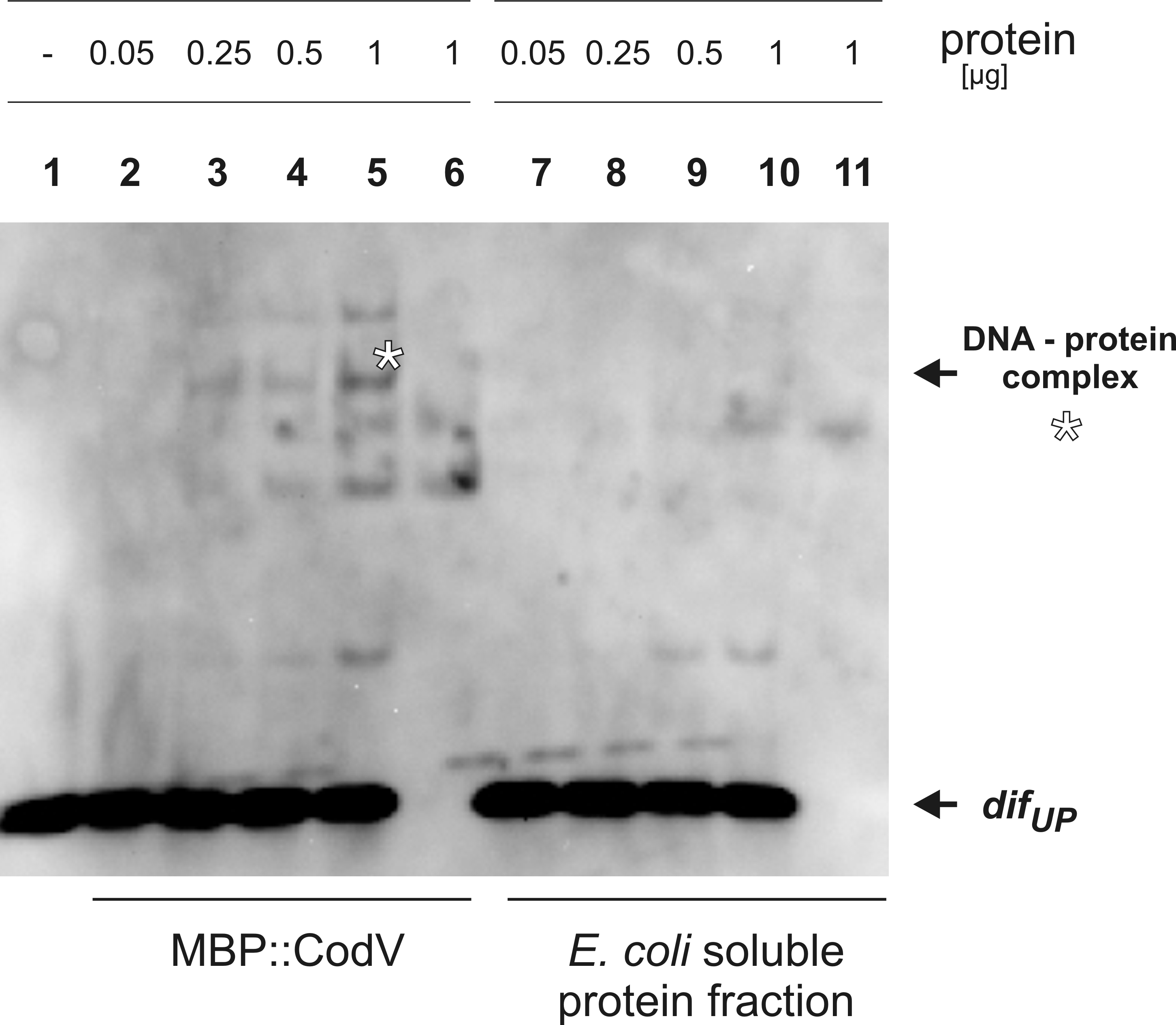


**Fig. S15. Protein concentration-dependent binding of MBP::CodV and the soluble fraction of *E. coli* DH10B to *dif_UP_*.**

EMSA analysis using purified MBP::CodV and the soluble protein fraction of *E. coli* DH10B at different concentrations and biotin labeled substrate *dif_UP_*.

Lane 1, *dif_UP_*

Lane 2, *dif_UP_* + MBP::CodV (50 ng)

Lane 3, *dif_UP_* + MBP::CodV(250 ng)

Lane 4, *dif_UP_* + MBP::CodV (500 ng)

Lane 5, *dif_UP_* + MBP::CodV (1 µg)

Lane 6, MBP::CodV (1µg)

Lane 7, *dif_UP_* + *E. coli* protein (50 ng)

Lane 8, *dif_UP_* + *E. coli* protein (250 ng)

Lane 9, *dif_UP_* + *E. coli* protein (500 ng)

Lane 10, *dif_UP_* + *E. coli* protein (1 µg)

Lane 11, *E. coli* protein (1 µg)
